# Modeling extrahepatic hepatitis E virus infection in induced human primary neurons

**DOI:** 10.1073/pnas.2411434121

**Published:** 2024-11-15

**Authors:** Michelle Jagst, André Gömer, Sanja Augustyniak, Mara Klöhn, Adriana Rehm, Rainer G. Ulrich, Verian Bader, Konstanze F. Winklhofer, Yannick Brüggemann, Ralf Gold, Barbara Gisevius, Daniel Todt, Eike Steinmann

**Affiliations:** ^a^Department for Molecular and Medical Virology, Institute for Hygiene and Microbiology, Ruhr University Bochum, Bochum 44801, Germany; ^b^Institute of Virology, University of Veterinary Medicine Hannover, Hannover 30559, Germany; ^c^Department of Neurology, St. Josef Hospital, Ruhr University Bochum, Bochum 44801, Germany; ^d^Friedrich-Loeffler-Institut, Institute of Novel and Emerging Infectious Diseases, Greifswald-Insel Riems 17493, Germany; ^e^German Centre for Infection Research, Partner site Hamburg-Lübeck-Borstel-Riems, Greifswald-Insel Riems 17493, Germany; ^f^Department of Molecular Cell Biology, Institute of Biochemistry and Pathobiochemistry, Ruhr University Bochum, Bochum 44801, Germany; ^g^European Virus Bioinformatics Center, Jena 07743, Germany; ^h^German Centre for Infection Research (DZIF), External Partner Site, Bochum 44801, Germany

**Keywords:** hepatitis E virus, primary neurons, human model system, innate immune responses, neurite outgrowth

## Abstract

Hepatitis E virus (HEV) infections are one of the most common causes of acute viral hepatitis, annually causing over 3 million symptomatic cases and 70,000 deaths worldwide. Historically, HEV was described as a hepatotropic virus, but has recently been associated with various extrahepatic manifestations including neurological disorders such as Guillain–Barré syndrome and neuralgic amyotrophy. However, the underlying pathogenesis of these neurological diseases remains largely unknown. The aim of this study was to investigate extrahepatic HEV manifestations in a neuronal model system using human-induced primary neurons (iPNs). Renal epithelial cells from human urine were reprogrammed to induced pluripotent stem cells to generate neuronal progenitor cells, which were subsequently differentiated into iPNs over 21 d. These iPNs supported HEV infection preferentially in neurite-bearing cells. Transcriptional profiling of the neuronal development process as well as viral infection dynamics in iPNs uncovered a lack of antiviral innate immune responses to HEV infection with only an intrinsic expression of distinct interferon-regulated genes and signaling molecules. Viral open reading frame 2 encoded capsid protein could be visualized by volumetric three-dimensional reconstitution within the neurites, which were reduced in length in an HEV inoculation-dependent manner. In conclusion, this neuron-derived human model system provides a powerful tool for studying extrahepatic manifestations of HEV infection. It further indicates a potential mechanism of pathogenesis driven by the interaction between host and viral factors.

## Significance

Hepatitis E virus (HEV) has increasing prevalence worldwide and is associated with extrahepatic manifestations, especially neurological disorders. However, the mechanisms by which HEV contributes to these diseases remain poorly understood. Here, we provide a model system of induced primary neurons (iPNs) that are susceptible to HEV infection. Our findings reveal that iPNs exhibit a lack of innate immune response to HEV and reduced expression of signaling genes. Importantly, we observed that HEV inoculation results in a significant reduction in neurite length in iPNs, uncovering a pathogenic mechanism in neuronal cells. This model system provides valuable insights into the neurotropism of HEV and offers a robust platform for further investigation of HEV-induced neurological disorders.

Hepatitis E virus (HEV) belongs to the family *Hepeviridae* and is characterized by a linear, positive-sense single-stranded RNA genome of ~7.2 kilobases (kb). Every year, HEV infects an estimate of 20 million people worldwide and is one of the most common causes of viral hepatitis (1). There are eight genotypes of the *Paslahepevirus balayani* species, four of which (HEV-1 to -4) cause the majority of human infections (2). HEV-1 predominantly circulates in Asian and African countries, while HEV-2 is most commonly found in Mexico and African countries. Both genotypes are spread via the fecal–oral route, while HEV-3 (mainly in Europe) and HEV-4 (mainly in China) are zoonotic and are most commonly transmitted through consumption of meat products from domestic pig or wild boar (3). In most cases, HEV infection results in a self-limiting acute course without any or only mild symptoms. However, pregnant women often present with severe disease progression when infected with HEV-1 or HEV-2 with a mortality rate of up to 30% (4, 5). Additionally, immunocompromised patients, such as solid organ transplant recipients or patients infected with the human immunodeficiency virus (HIV), are at high risk of suffering a chronic HEV-3 infection which could promote liver fibrosis or cirrhosis (6, 7). However, treatment options remain limited for patients with chronic HEV infection as there is no antiviral specifically targeting HEV directly. Available therapies include the off-label use of ribavirin (RBV).

In recent years, increasing evidence has been provided that HEV can lead to extrahepatic manifestations such as glomerulonephritis, cryoglobulinemia, acute pancreatitis, hematological diseases, thyroiditis, and myocarditis (8, 9). Moreover, neurological diseases especially of the peripheral nervous system (PNS), such as Bell´s palsy, Guillain–Barré syndrome (GBS), and neuralgic amyotrophy (NA), have been found to correlate with HEV infections (10). For NA in particular, several clinical studies have already been conducted in different European countries (e.g., the Netherlands, Germany, Switzerland, France/UK/Belgium) showing that up to 11% of neurologically affected patients were associated with HEV infection (11–14). Notably, in the Netherlands, 10 out of 201 GBS patients showed an increased ratio of anti-HEV immunoglobulin (Ig) M antibodies (15). Other studies from Japan, Belgium, and Bangladesh documented 3.2% (2/63), 8% (6/73), and 11% (11/100) anti-HEV IgM-positivity in GBS patients, respectively (16–18). The majority of neurological disorders were associated with HEV-3 infection, but cases of HEV-1 or HEV-4 infection have also been described (10). Intriguingly, extrahepatic complications have been reported in both acutely and chronically infected individuals (8). In addition, HEV RNA has been detected in cerebrospinal fluid (CSF) of patients with chronic infection and phylogenetic analyses have provided evidence of HEV compartmentalization (19–21). To date, it remains unclear whether extrahepatic manifestations, particularly neurological disorders, are caused by direct HEV replication in affected tissues or by indirect immunopathological mechanisms. The most discussed indirect immunopathological mechanism for HEV-induced neurological disorders is molecular mimicry, where viral epitopes cross-react with self-antigens. Although this has not yet been proven for HEV, it remains a plausible explanation, especially for GBS associated with HEV, as this mechanism has been reported in cases of GBS induced by bacterial or other viral infections. Recent data have shown that HEV is able to cross the blood–brain barrier in vitro probably by infecting microvascular endothelial cells and that HEV can infect and replicate within neuronal-derived cancer cells in vitro (22–24). However, the pathogenesis of these extrahepatic manifestations is still largely unknown.

In this study, we aimed to understand the relationship between HEV and neurological manifestations by implementing and characterizing a primary neuronal model system for HEV infection.

## Results

### Human-Specific Induced Primary Neurons (iPNs) Express Markers of Mature Neurons.

**Fig. 1.**
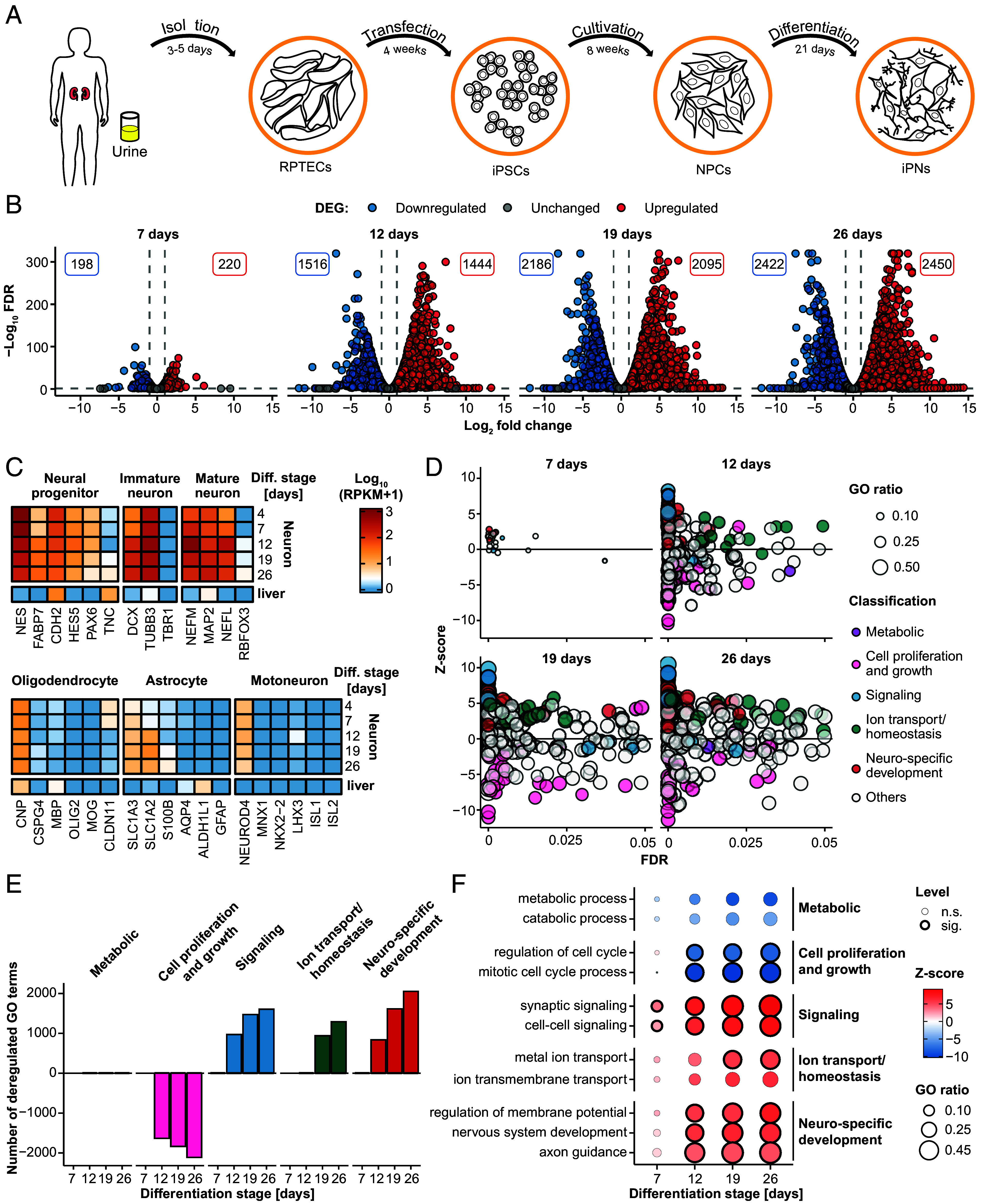
Characterization of induced human primary neurons as a model system for HEV. (*A*) First, RPTECs were isolated from human urine. The RPTECs were transfected with episomal vectors to iPSCs and further cultivation resulted in NPCs. iPNs were generated by differentiating the NPCs over 21 d. (*B*–*F*) Transcriptome analysis (Illumina sequencing) during the differentiation process with comparison of cells aged 7, 12, 19, and 26 d to the earliest time point of 4-d-old cells. (*B*) Volcano plots show the strength of deregulation during cell differentiation. Total number of significantly DEG, up-regulated (red), or down-regulated (blue), at different stages of differentiation. The threshold for DEG was set at a FC above 4 or below -4, a FDR with a minimum of 0.05 and RPKM of at least 0.5. (*C*) Expression pattern of marker genes for neuronal progenitors, immature neurons, mature neurons, oligodendrocytes, astrocytes, and motoneurons during the differentiation process. Color-code represents the log_10_ (RPKM+1) values. (*D*) Overview of the significantly deregulated GO terms classified into metabolic (purple), cell proliferation and growth (pink), signaling (blue), ion transport/homeostasis (green), and neuro-specific development (red) processes. The z-score (y-axis) indicates the activation or deactivation of GO terms and the dot size depicts the fraction of deregulated genes per term. (*E*) Quantification of significantly deregulated GO terms associated with metabolic processes, cell proliferation, signaling pathways, ion transport/homeostasis, and neuro-specific development compared across different stages of cellular differentiation. Threshold for GO term activation or deactivation was set at *P* value <= 0.05 and term size of at least 30 genes. (*F*) Selected GO terms, encompassing metabolic processes, cell proliferation and growth, signaling pathways, ion transport/homeostasis, and neuro-specific development, were investigated across various time points of differentiation. The dot color reflects whether a term is activated or deactivated (z-score). The GO ratio indicates the number of deregulated genes per term and the dot border is a binary indicator for a significant (FDR < = 0.05) deregulation. n.s: not significant; sig: significant.

To understand how HEV infection leads to neurological diseases, we implemented a cell culture model system of iPNs from human specimen (Fig. 1*A*) (25). To generate iPNs, renal proximal tubule epithelial cells (RPTECs) were isolated from a urine sample of a healthy individual (Fig. 1*A*). Plasmid-mediated transfection with pluripotency-inducing genes (*Oct3/4*, *Nanog*, *c-Myc*, *Klf4*) led to the reprogramming of RPTECs and the generation of induced pluripotent stem cells (iPSCs). Embryoid bodies were formed via cell aggregation, accompanied by the suppression of meso- and entodermal lineages, ultimately resulting in the predominance of an ectodermal lineage. Afterward, embryoid bodies were cultivated under adherent conditions and in the presence of epidermal growth factor (EGF) and fibroblast growth factor (FGF) to facilitate the formation of neural rosettes. Neural rosettes were subsequently isolated and dissociated into neural progenitor cells (NPCs). These cells were then differentiated into iPNs by addition of basal medium supplemented with sonic hedgehog (SHH), retinoic acid (RA) (days 1 to 6) for differentiation and brain-derived neurotrophic factor (BDNF) and glial cell line-derived neurotrophic factor (GDNF; from day 7 onward) for maturation over a period of 21 d (Fig. 1*A*). To characterize the final steps of the differentiation process from NPCs, we isolated cellular RNA at different time points (4, 7, 12, 19, and 26 d) and performed transcriptional profiling by RNA-sequencing (Fig. 1*B*). Throughout the differentiation process, there was an increase in the quantity of deregulated genes. At 7 d of maturation, neurons exhibited approximately 200 genes that were up- and down-regulated compared to 4-d-old cells, while this number increased to ~2,400 down- and up-regulated genes by the 26th day of differentiation (Fig. 1*B*). There was a significant degree of overlap between the number of genes that were similarly deregulated at days 12, 19, and 26 (1,172 up- and 1,101 down-regulated) (*SI Appendix*, Fig. S1*A*). As depicted in Fig. 1*C*, the expression levels of marker genes associated with NPCs, immature neurons, and mature neurons remained consistently high throughout all assessed time points during the process of cellular differentiation. In contrast, expression of gene markers for oligodendrocytes, astrocytes, and motoneurons, remained low, indicating a primarily neuron cell culture (Fig. 1*C*). Gene ontology (GO) analysis revealed a noticeable similarity between 7-d-old neurons and the early differentiation stage of 4 d, while after 12 and 19 d of maturation around 400 GO terms were differentially regulated (Fig. 1*D* and *SI Appendix*, Fig. S1*B*). Most of the deregulated GO terms belonged to signaling, ion transport/homeostasis, and neuro-specific development processes. The positive z-score, in conjunction with the high number of differentially regulated genes within these terms (shown as dot size), suggests that the majority of the genes included in these GO terms were up-regulated (Fig. 1 *D* and *E*). In particular, the GO terms nervous system development, axon guidance, regulation of membrane potential, synaptic signaling as well as cell–cell signaling were significantly up-regulated, while cell proliferation and growth and metabolic processes showed after 12 d of differentiation a negative z-score, indicating a downregulation (Fig. 1 *D* and *F*).

In summary, functional human-specific iPNs were generated and expressed respective markers of mature neurons. Throughout the differentiation of NPCs, we observed an increasing number of deregulated genes, which were mainly involved in neuro-specific developmental processes and signaling pathways.

### iPNs Are Susceptible to Cell Culture-Derived HEV.

**Fig. 2.**
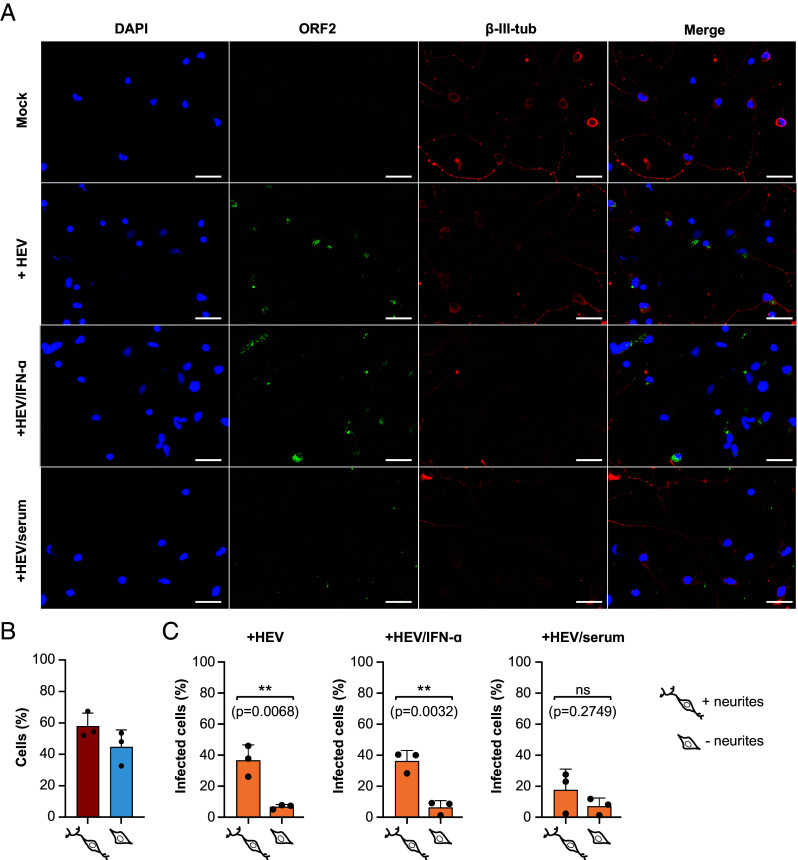
Infection of human-iPNs with HEVcc Kernow-C1 p6 strain. (*A*) Immunofluorescence staining was performed for uninfected (Mock) and infected iPNs without treatment or with IFN-α (1,000 Units/mL) or human anti-HEV serum. DAPI was used to stain the nuclei (blue), a polyclonal rabbit anti-HEV antiserum to stain the ORF2-encoded capsid protein (green) and an anti-β-III-tubulin (β-III-Tub) antibody to stain the neuronal cytoskeleton. (Scale bars represent 50 µm.) (*B*) The percentage of neurons with or without neurites were determined by quantifying the β-III-tubulin signal. (*C*) The susceptibility of neurons, both with and without neurites, to HEV infection was quantified by measuring the ORF2 signal using CellProfiler. An unpaired *t* test was used to assess statistical significance with a significance level of α = 0.05. ns: not significant.

Next, we assessed the susceptibility of iPNs (21 d old) to cell culture-derived naked and quasi-enveloped HEV-3 (HEVcc and eHEVcc) Kernow-C1 p6 strain as well as to the HEV-3 wild boar strain 83-2 (26, 27). At 5 d postinfection immunofluorescence staining was conducted against the open reading frame 2 (ORF2)-encoded capsid protein of HEV and a specific neuronal cytoskeleton marker (β-III-tubulin) (Fig. 2). Primary neurons could be efficiently infected with HEVcc, eHEVcc, and the HEV-3 strain 83-2 (Fig. 2*A* and *SI Appendix*, Fig. S2 *A* and *B*). Further, HEVcc infection was blocked by a human anti-HEV serum, but not by interferon alpha (IFN-α) (Fig. 2*A*). A CellProfiler pipeline was employed to ascertain the presence and length of neurites via β-III-tubulin immunofluorescence staining and the HEV infection status of cells by immunofluorescence staining of the capsid protein. This analysis revealed that cells possessing neurites exhibited increased susceptibility to HEV, with over 30% of cells with neurites infected compared to 10% of cells without neurites infected (Fig. 2 *B* and *C*). The same trend was observed for IFN-α or anti-HEV serum-treated cells. To visualize the infection process of iPNs in more detail, the subcellular distribution of ORF2-encoded capsid protein and cellular marker proteins (cytoskeleton, Golgi apparatus, and the endoplasmic reticulum) was assessed by immunofluorescence staining with corresponding antibodies. As depicted in *SI Appendix*, Fig. S3*A*, the viral ORF2 antigen could be detected in the perinuclear region with the highest colocalization with the Golgi marker (GM130) (*SI Appendix*, Fig. S3 *A* and *B*). Additionally, volumetric three-dimensional reconstitution showed clusters of ORF2 in the neurites, as well as around and within the nucleus (*SI Appendix*, Fig. S3*C* and Movie S1). To conclude, iPNs were susceptible to HEVcc and could therefore serve as a human model system for studying the neurotropism of HEV.

### Differentially Primary Neurons Challenged with HEVcc Lack Antiviral Immune Responses.

**Fig. 3.**
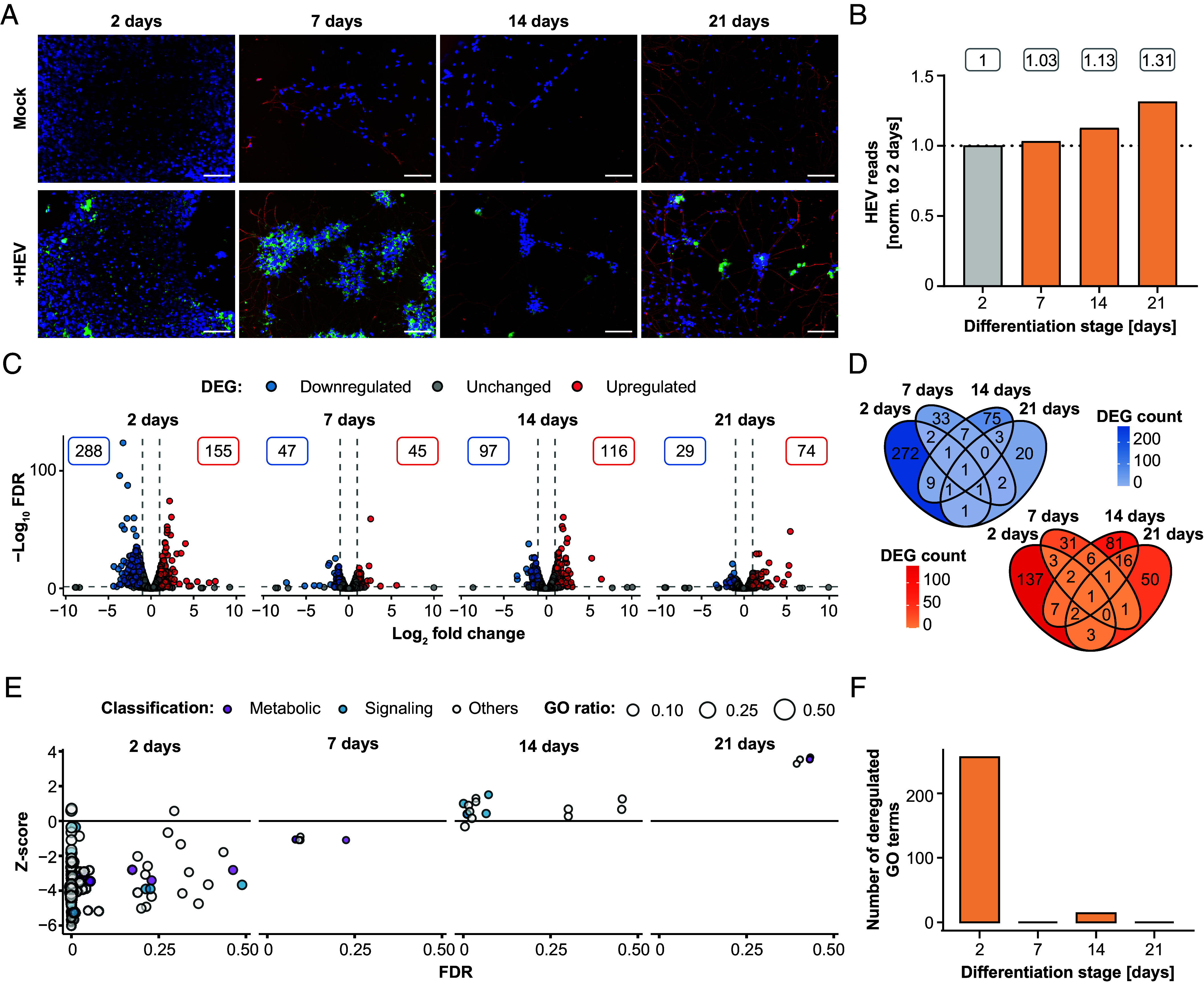
RNA sequencing analysis of cellular response to HEV infection in iPNs. (*A*) Along the differentiation process from neural progenitor cells to mature neurons, differentiating cells were infected with HEV at defined time points of 2, 7, 14, and 21 d after the initiation of the differentiation process. Five-days postinoculation, immunofluorescence staining was performed for uninfected (Mock) and infected (+HEV) cells using a polyclonal rabbit anti-HEV antibody for the ORF2 capsid protein (green), β-III-tubulin for the cytoskeleton (red) and DAPI for the nucleus (blue). Scale bars represent 100 µm. (*B*–*F*) Illumina RNA sequencing analysis was conducted on samples obtained from both uninfected and HEV-infected cells after a 5-d infection period, encompassing various differentiation stages (2, 7, 14, 21 d), followed by comparative assessment at each time point to elucidate differences in gene expression profiles between uninfected and HEV-infected neurons. (*B*) The depicted values represent the FC of HEV reads, normalized to total reads, across different stages of differentiation normalized to cells aged 2 d. (*C*) Volcano plots show the strength of deregulation under HEV infection with the total number of significantly DEG, up-regulated (red), or down-regulated (blue). The threshold for DEG was set at an absolute FC of 2, a FDR < 0.05 and max group expression of 2 transcript per million. (*D*) Venn diagram of significantly up-regulated (red) and down-regulated DEGs (blue) through HEV infection. (*E*) Overview of deregulated GO terms classified into metabolic (purple) and signaling (blue) processes. The z-score (*y*-axis) shows the up- or downregulation and dot size indicates the number of DEGs per term. (*F*) Quantification of the significantly deregulated GO terms with an FDR under 0.05 for each time point.

**Fig. 4.**
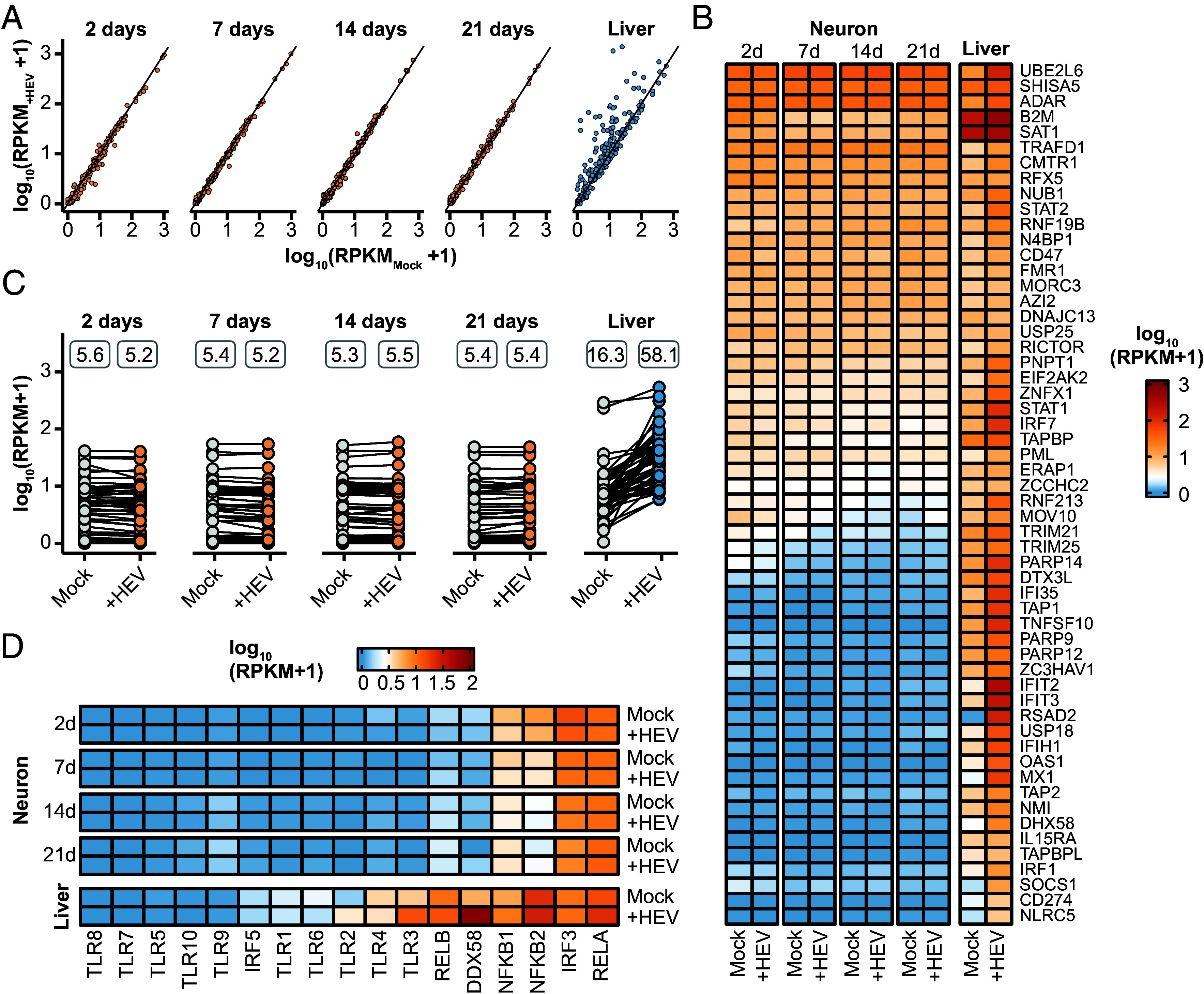
Neuronal intrinsic expression of ISGs and absence of innate immune response to HEV infection. (*A*) Overview of the general immune response. Depicted are all genes within the GO term immune response (GO:0006955) of uninfected (*x*-axis) and infected (*y*-axis) neurons at different differentiation time points (2, 7, 14, 21 d) and primary human hepatocytes (PHH, liver). The primary neurons (orange) were infected with HEV for 5 d and the PHHs (blue) were infected for 48 h. (*B*) Expression pattern of core ISGs of uninfected (Mock) and infected (+HEV) primary neurons at different differentiation stages and PHH samples. Color-code represents log_10_ RPKM+1 values. (*C*) Overview and comparative analysis of the expression levels of core ISGs measured in RPKM values, in both uninfected (Mock) and HEV-infected (+HEV) primary neurons and PHH. The values above displayed represent the mean RPKM expression levels of all genes under each respective condition. (*D*) Expression pattern of pathogen-sensing genes including Toll-like receptors of Mock and HEV-infected iPNs and PHH.

To explore HEV-specific responses in gene expression in the various differentiation stages of NPCs to mature neurons (2, 7, 14, 21 d), RNA sequencing was conducted 5 d postinfection. Then, time point-matched statistical analysis was performed to characterize changes in gene expression pattern between infected and noninfected cells. Brightfield imaging across three differentiation timepoints revealed a progression from an absence or minimal presence of neurites at day 0 to an average of 2-4 neurites per cell following 21 d of differentiation (*SI Appendix*, Fig. S4). At all tested time points of differentiation, iPNs were susceptible to HEVcc as demonstrated by immunofluorescence staining (Fig. 3*A*). Subsequently, we quantified the total number of viral reads as proxy for HEV replication from RNA sequencing data, which showed an increase over the differentiation period, with the highest levels observed at days 14 and 21 (Fig. 3*B* and *SI Appendix*, Fig. S5*A*). In line with previous findings by Todt et al. (27), subgenomic HEV transcripts with overlapping ORF2/ORF3 were more abundant than ORF1-containing genomic viral RNA (*SI Appendix*, Fig. S5*A*). The reads that mapped to the viral genome exhibited peaks of coverage in the mock control, resembling mismatches at the locus of the human S17 insertion on the hypervariable region of the Kernow-C1 p6 strain (28) (*SI Appendix*, Fig. S5*A*). Analysis of significant differentially expressed genes (DEGs) in infected iPNs compared to uninfected cells revealed distinct expression patterns for each time point of differentiation with minimal overlap (Fig. 3 *C* and *D*). The strongest induction of gene expression was present in cells 2 d after initiation of differentiation. Followingly, the number of DEGs was found to be reduced with the time of differentiation. Comparison of the identified DEGs with previously described interferon (IFN)-regulated genes (IRGs) (29) revealed only a few significantly up- or down-regulated IRGs (*SI Appendix*, Fig. S5*B*) implying a lack of innate immune responses 5 d postinfection during the differentiation process of iPNs. These findings were further supported by GO analysis, which revealed that only in 2-d-old primary neurons more than 200 GO terms were significantly deregulated, most of them showing a negative z-score and belonging to metabolic or signaling processes (Fig. 3 *E* and *F*). In contrast, at day 7 and 14, no GO terms were significantly deregulated in HEV-infected cells, indicating a weak to no deregulation of pathogen-mediated cellular gene expression (Fig. 3*F*). Subsequently, in order to gain a more comprehensive understanding of the absence of significant alterations in transcriptomic responses to infection, the expression of genes that participate in the innate immune response was analyzed in iPNs and in primary human hepatocytes (PHH). A comparison to the host innate immune responses in the liver showed distinct antiviral response signatures between neuronal and liver cells (Fig. 4). While the expression of innate immune system genes (GO:0006955) was not altered in HEV-infected iPNs, their expression was found to be increased in PHHs following infection (Fig. 4*A*). Furthermore, analysis of the expression levels of interferon-stimulated genes (ISGs) in iPNs and PHHs demonstrated an intrinsic expression of ISGs in iPNs, including double-stranded RNA-specific adenosine deaminase (*ADAR*) and signal transducer and activator of transcription 1 and 2 (*STAT1*, and *STAT2*), although at a lower level than in PHH. However, in contrast to PHHs, the expression of ISGs did not change upon infection, regardless of the differentiation stage of the neuronal cells (Fig. 4 *B* and *C*). Subsequently, we wanted to prove whether trends in baseline ISG expression similar to those seen in iPNs and PHHs can be observed in publicly available single-cell RNA sequencing data from neuronal and liver tissue biopsies. This analysis confirmed that neurons exhibited an intrinsic ISG expression, which was reduced for oligodendrocytes or astrocytes (*SI Appendix*, Fig. S6*A*). Given that the absence of robust innate immune responses may be associated with the expression of pattern recognition receptors, their expression was analyzed. Among antiviral signaling molecules, only interferon regulatory factor 3 (*IRF3*) and the nuclear factor-kappaB subunit *RELA* displayed high expression levels, whereas genes of toll-like receptors (TLRs) were not detected (Fig. 4*D* and *SI Appendix*, Fig. S6*B*). A similar trend was substantiated by single-cell RNA sequencing data, which revealed generally low to undetectable expression levels of TLRs in neurons (*SI Appendix*, Fig. S6*C*).

In summary, these results demonstrate increased HEV infection level during iPN differentiation and a lack of innate immune responses to viral infection in neuronal cells.

### Neurite Length Difference of iPNs after HEV Inoculation.

**Fig. 5.**
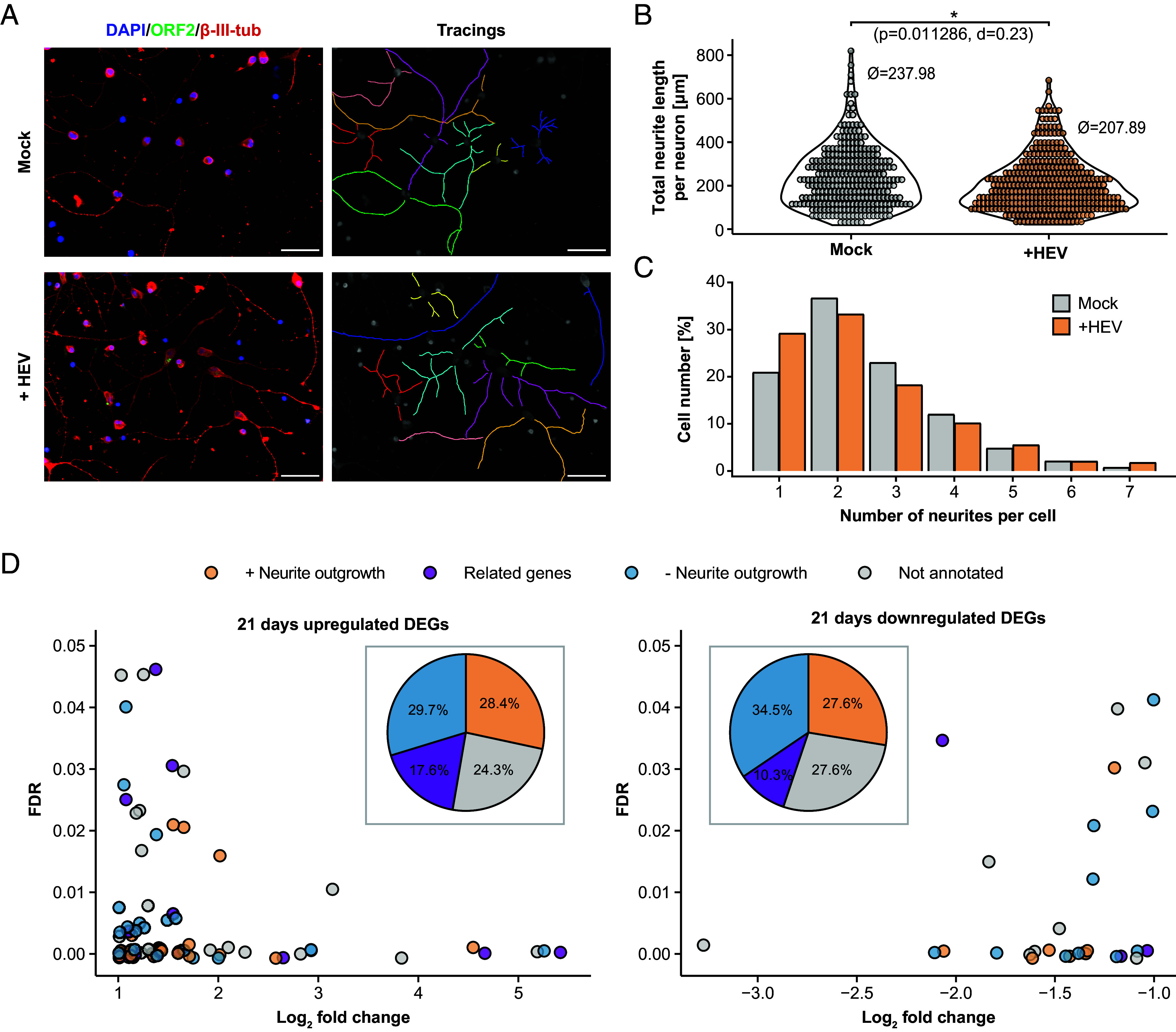
Reduction of neurite length of iPNs after HEV-inoculation. The effect of HEV inoculation on iPNs neurite length compared to mock-inoculated cells (21-d-old cells) was examined through microscopic analysis. Five days postinoculation, cells were stained with DAPI (blue, nucleus) and with antibodies against the ORF2 capsid protein (green) and β-III-tubulin (red, cytoskeleton). ImageJ’s NeuronJ plugin was used to manually track the neurites of each neuron (*A*) and determine the total length of neurites per neuron (*B*) and the number of neurites per cell (*C*). To test the significance of differences in neurite length, Mann–Whitney *U* test followed by Bonferroni *P*-value correction was used. The effect size was calculated as Cohen´s d (d). (Scale bars represent 100 µm.) (*D*) A literature review was conducted on the 72 up- and 29 down-regulated genes identified in 21-d-old HEV-infected cells to determine whether these genes were previously reported in connection with neurite outgrowth (orange), had related genes (family members) associated with neurite outgrowth (purple), had no known relation to neurite outgrowth, or were not annotated (*SI Appendix*, Tables S1 and S2). The pie chart represents the percentage of these categories relative to the total number of significantly up- and down-regulated genes.

Next, we used the model system of iPNs to analyze the neurite growth by tracing neurite length in HEV-inoculated and mock-inoculated 21-d-old neurons, with analysis conducted 5 d postinfection (Fig. 5*A*). A significant reduction of the average neurite length was observed of neurons inoculated with HEV, with the total neurite length decreasing by 30 µm (Fig. 5*B*). Further, a shift to shorter neurites was observed, indicating a higher proportion of neurons with reduced neurite length after HEV inoculation (*SI Appendix*, Fig. S7). At the same time, HEV inoculation did not impact the number of neurites per cell, as evidenced by the consistent observation of predominately 1 or 2 neurites per neuron in both control and HEV-inoculated cells (Fig. 5*C*). To better understand this phenomenon, the set of 74 up-regulated and 29 down-regulated genes after HEV inoculation identified in neurons aged 21 d (Fig. 3*C*) were analyzed for their association with neurite outgrowth. For each of these genes, the expression pattern differs across the differentiation stages. For example, vascular endothelial growth factor A (*VEGFA*) and caveolin 1 (*CAV1*), both known to impact neurite outgrowth, were only up-regulated at the latest differentiation time point of 21 d (*SI Appendix*, Fig. S8). Among the genes that were significantly deregulated were MOV10 RISC complex RNA helicase (*MOV10*), activating transcription factor 3 (*ATF3*), three prime repair exonuclease 1 (*TREX1*), ArfGAP with RhoGAP domain, ankyrin repeat and PH domain 3 (*ARAP3*), brevican (*BCAN*), Purkinje cell protein 4 like 1 (*PCP4L1*), and regulator of G protein signaling 5 (*RGS5*), which are associated with neurite outgrowth (*SI Appendix*, Tables S1 and S2). Notably, certain members of deregulated gene sets, including serpin family E member 1 (*SERPINE1*), plasminogen activator, urokinase (*PLAU*), and plasminogen activator, urokinase receptor (*PLAUR*), are associated with the brain plasminogen activating system, and seems to play a role in neurite outgrowth. Furthermore, alterations were observed in transforming growth factor beta induced (*TGFBI*) and endoglin (*ENG*), integral components of the TGF-beta system (*SI Appendix*, Tables S1 and S2). Literature research revealed that 28.4% and 27.6% of the up- and down-regulated genes, respectively, had been previously linked to neurite outgrowth, while 17.6% and 10.3% of the genes had associated genes known to be involved in neurite outgrowth indicating that potentially HEV can affect the neurite length of iPNs (Fig. 5*D*).

Overall, we observed a significant reduction of the neurite length after HEV-inoculation in iPNs, with a substantial number of significant deregulated genes known to influence neurite length.

## Discussion

HEV-infected patients can suffer from extrahepatic manifestation, which include mainly neurological sequelae such as GBS, NA, or meningoencephalitis (10, 30). One hypothesis proposes direct infection of human neuronal cells, supported by in vitro evidence of HEV replication in human neuronal-derived cells (22, 23, 31). Further, HEV RNA was found in the CSF of multiple HEV-infected patients and recently in the medulla oblongata in the human brain (19, 21, 31, 32). Additionally, Tian et al. demonstrated HEV’s ability to cross the blood–brain barrier by infecting endothelial cells in vitro providing a potential entry route to the CNS (24). However, the precise mechanism by which HEV induces neurological symptoms in humans and the possible interplay between neuronal host cells and virus remains unclear, emphasizing the urgent need to better understand extrahepatic phenomena.

In this study, we present a model system enabling the in vitro investigation of the interactions between HEV and human-iPNs. The model system is based on a patient-friendly noninvasive acquisition of human material via urine samples, where the RPTECs could be isolated and reprogrammed to generate iPSCs (25). This method of acquiring patient material eliminates the need for invasive medical procedures such as skin punches or surgeries. Additionally, it enables the generation of personalized in vitro systems, allowing the consideration of genetic and epigenetic variation of the host on HEV infection, thereby facilitating patient-specific treatment and risk assessment. A limitation of this system is the time required to process the urine sample to obtain primary neurons, making it especially suitable as a model system for chronically infected patients, as acutely infected patients either resolve the infection or become chronic before iPNs are available. Here, the iPSCs were differentiated into iPNs that highly expressed marker genes of neuronal progenitor, immature, and mature neurons. These neurons exhibited susceptibility to the HEV-3 Kernow-C1 p6 strain, with a notably higher infection rate of approximately 30% observed in neurons possessing neurites compared to those lacking neurites. The reason for this phenomenon could be that neurites-bearing cells have a higher density of an unknown receptor or that viruses reach the neurites before the cells bearing no neurites. Future studies are needed to mechanistically decipher this observation. Moreover, early stages of the differentiation process from NPCs to mature neurons were also susceptible to HEV, while the number of HEV reads increased throughout differentiation, indicating an enhanced replication capacity in differentiated neurons. Most cases of neurological diseases in connection with HEV are reported with HEV-3, but cases of HEV-1 and HEV-4 have also been described (10). Due to lack of efficient cell culture systems of other genotypes, we tested only strains of HEV-3. Next, we studied in detail the host-induced reaction to viral infection on a transcriptomic level, seeing that iPNs have an intrinsic expression of the core ISGs like ADAR, STAT1, STAT2, or CD47, which was confirmed with publicly available single-cell datasets. The infection with HEV did not induce significant alterations in the expression levels of ISGs within neurons, unlike PHH, where the activation of innate immunity via interferons occurs. It is well known from hepatoma cells, that HEV is recognized via pattern recognition receptors (PRRs) like RIG-I (DDX58) or TLR3, which activates the cascade of interferon signaling and provides immune responses to eliminate pathogens (27, 33). The nervous system however, especially the CNS, is immune privileged. Here, strong immune responses may be triggered by microglial or astrocytes which can be activated through the secretion of neuropeptides or chemokines by neurons (34, 35). To the best of our knowledge, the susceptibility of nervous system immune cells (e.g., microglia and astrocytes) to HEV and their potential response to the virus remain unknown. Investigating these aspects could provide valuable insights for future studies. In our system, HEV may not be sensed via PRRs, as only IRF3 and RELA genes exhibited high expression levels. This is in line with described expression pattern of TLRs, which showed in general low expression in neurons, especially when compared to microglia or astrocytes (36, 37). However, in mouse primary neurons, it was demonstrated that Zika virus can induce an IFN-beta response, albeit with delayed activation (38). Another aspect described for certain neurotropic viruses, including Zika, Rabies, HIV, and Herpes Simplex Virus (HSV), is their impact on neuronal morphogenesis and neurite outgrowth (39–42). For example, HSV is able to manipulate the protein composition of the secreted vesicles of infected endothelial cells via the glycoprotein which lead to an increase of neurite length in neurons and therefore facilitate neuronal infection (42). Understanding of the neuropathological effects induced by HEV remains limited. HEV-4 RNA and ORF2-encoded capsid protein was found in the brain and spinal cord of infected rabbits and Mongolian gerbils, where CNS pathologies like perineural invasion, neuron necrosis, microglia nodule, lymphocyte infiltration, perivascular cuff, and myelin degeneration were observed (43–45). In addition, these studies reported potential damage to the blood–brain barrier, as reduced levels of tight junction proteins including Claudin-5, Occludin, and ZO-1 were observed in these animal models. In this study, neurite length was tracked under both mock-inoculated and HEV-inoculated conditions, revealing a reduction in neurite outgrowth after HEV inoculation. One potential mechanism by which HEV may affect neurite outgrowth is through modulation of the host transcriptome. This could be supported by our finding that, numerous factors previously implicated in neurite outgrowth demonstrated significant up- or downregulation in the differentiated primary cells, constituting approximately 30% of all deregulated genes. It is also possible that HEV inoculation may have resulted in a diminished regenerative capacity in iPNs, consequently affecting neurite length. The number of neurites per cells, however, remains unaffected by virus infection. At present, we can demonstrate the effect of neurite length modulation by HEV infection, but do not know whether this is a viral-driven or indirect immune-driven mechanism. In follow-up studies, one could express single HEV proteins in iPNs and test whether RNA replication is required for the length differences. Another approach could be to differentiate these cells from urine of different acute and chronic HEV patients to investigate this effect in a disease model.

All in all, we provide a patient-specific model system to study the extrahepatic manifestation of HEV infection and, in particular, the interplay between neurons and HEV. We demonstrated a lack of innate immunity in neurons and a decrease in neurite length after HEV inoculation.

## Material and Methods

### Generation of iPNs.

iPNs were generated accordingly to the protocol of Massa et al. (25). In fact, RPTECs were isolated from human urine of a healthy patient (Reg. Nr. 4493-12). The human samples were deidentified before use. RPTECs were cultivated in RGEM medium (#CC-3190, Lonza) until the reprogramming to iPSCs. Hence, a reprogramming cassette comprising pCXLE-hOCT3/4-shp53 (#27077), pCXLE-hSK (#27078), and pCXLE-hUL (#27080) plasmids sourced from Addgene was transfected utilizing the Neon® Transfection System (Life Technologies) (46). Colony formation of cells was ensured by cultivation of reprogrammes RPTECs in TeSR™-E7™ medium (#05914, Stemcell Technologies). In the following, iPSCs-colonies were cultivated in mTeSR™1 medium (#85850, Stemcell Technologies). To initiate embryoid body formation, iPSCs were cultured under nonadherend conditions in mTeSR™1 medium. The development of the mesoderm and ectoderm lineage was inhibited by administration of 10 µM SB431542 (#FBM-10-2443, Biozol) and 5 µM Dorsomorphin (#866405-64-3, Sigma). After 6 d, embryoid bodies were transferred onto 0.002% poly-L-ornithine (PORN, #27378-49-0, Sigma)/10 µg/mL laminin (#114956-81-9, Sigma) coated dishes and were cultured in neural stem cell medium (NSCM), composed of Dulbecco’s modified Eagle’s medium (DMEM)/F12 GlutaMAX™ (#31331028, Thermo Fisher Scientific) supplemented with 20 µg/mL insulin (#91077C, Sigma), 1.6 g/L L-glucose (Applichem), 1 µL/mL B-27TM (#17504-044, Life Technologies), 1 µL/mL N-2 (#17502048, Life Technologies), 10 ng/mL bFGF (#1102021, PAN Biotech), 10 ng/mL EGF (#1101001, PAN Biotech). Cultivation of adherent embryoid bodies in NSCM leads to the development of neuroectoderm and subsequently to the formation of neural rosette structures. Once neuronal rosettes were formed, structures were manually dissected via trypsinization for the isolation of neuronal progenitor cells (NPCs), which were then cultivated in NSCM. Next, NPCs were differentiated into iPNs by cultivation in DMEM/F12 GlutaMAX™ supplemented with 2× N-2, 2× B-27™, 50 µg/mL apo-transferrin (#T8158, Sigma), and 200 µg/mL L-ascorbic acid (#3525.1, Carl Roth). Differentiation was thereby induced by administration of 500 ng/mL of sonic hedgehog (#100-45, PeproTech) and 4 µM of retinoic acid (#R2625, Sigma); maturation and maintenance was achieved by supplementing 10 ng/mL of brain-derived neurotrophic factor (BDNF, #450-02, PeproTech) and 20 ng/mL of glial cell-derived neurotrophic factor (GDNF, #450-10, PeproTech) after day 6 of differentiation. Cells were incubated at 37 °C, 5% CO_2_ content, and with around 95% humidity. Type-I interferon α-2a (ProSpec; 1,000 Units/mL) was used in the infection experiment in iPNs with a concentration of 1,000 Units/mL.

### HEV Constructs and In Vitro Transcription.

In order to produce HEVcc, a plasmid construct of full-length HEV p6 clone (Kernow-C1; genotype HEV-3; GenBank accession number JQ679013) and a full-length HEV wild boar 83-2-27 strain (genotype HEV-3; GenBank accession number AB740232) was used and in vitro transcribed. For that, the plasmid was first linearized using the enzyme MluI enzyme and purified using the Qiaquick Spin Mini Kit (Qiagen), followed by in vitro transcription as described previously (27, 47). In brief, a mix containing 2 µg linearized plasmid, 5 mM Ribo m7G Cap analogue (Promega), and 4 µL T7 RNA polymerase were incubated at 37 °C for 4 h, with 2 µL of T7 RNA polymerase replenished after the first 2 h. Subsequently, the DNA template was digested by adding 7.5 µL DNase (Promega) and RNA was purified using the NucleoSpin^®^ RNA Clean-up Kit (Macherey-Nagel).

### HEV Infection and Immunofluorescence Staining.

Virus particles produced in hepatic HepG2 cells as previously described, were used with a multiplicity of infection (MOI) of 10-40 to infect 2 d, 7 d, 14 d, and 21 d old cells during the differentiation from NPCs to iPNs (47). Five days postinfection, cells were fixed with 3% paraformaldehyde (PFA) in phosphate-buffered saline (PBS) for at least 30 min, washed with PBS, and were permeabilized with 0.2% Triton-X 100 in PBS. After another washing step with PBS, the samples were blocked with 5% horse serum (HS) for at least 1 h and then cells were incubated with the first antibody (β-III-tubulin (1:20,000; mouse; Invitrogen), Calreticulin (1:200; rabbit; Cell Signaling Technologies), α-tubulin (1:1,000; mouse; Santa Cruz), or GM130 (1:400; rabbit; Cell Signaling Technologies) overnight. A polyclonal HEV-3 capsid protein-specific rabbit hyperimmune serum (diluted 1:5,000 in 5% horse serum) was used to stain for the ORF2-encoded capsid protein. On the next day, the cells were incubated for 2 h with the secondary antibody (goat anti-rabbit AlexaFluor 488 or donkey-anti-mouse AlexaFluor 555, 1:1,000 in 5% horse serum, Invitrogen). Finally, the nuclei were stained with DAPI (1:10,000 in H_2_O, Invitrogen). Images were taken with a Keyence BZX800 microscope with 20×, 40×, or 60× objectives.

### Iterative Indirect Immunofluorescence Imaging.

To study colocalization between proteins and cellular components in iPNs, we used iterative indirect immunofluorescence imaging (48), which allows staining of the same cells with different antibodies in multiple rounds. iPNs were seeded in an 8-well plate (Ibidi) and 5 d postinfection, iPNs were washed once with PBS and were then fixed with 3% PFA. Next, the cells were washed again with PBS and were permeabilized with 0.2% Triton-X 100, allowing subsequent antibodies to penetrate the cells. Unspecific binding sides were blocked with blocking solution containing 2% albumin fraction V pH 7.0 (BSA) (AppliChem), 200 mM ammonium chloride, and 300 mM maleimide (Sigma-Aldrich) in PBS for 1 h on a shaker. After another washing step with PBS, iPNs were incubated with the respective primary antibody overnight, followed by an incubation with secondary antibody for 2 h. Images were acquired, and the antibodies eluted. For this, cells were incubated with elution buffer (adjusted to pH 2.5) containing L-glycine (0.5 M), urea (3 M), guanidine HCl (3 M), and Tris(2- carboxyethyl)phosphine hydrochloride (70 mM) (Sigma-Aldrich) on a shaker for 10 min. Last, new antibodies were used for further rounds of staining. Colocalization between ORF2 signal and GM130 (Golgi apparatus) signal was analyzed using the ‘Plot Profile’ tool from the image analysis software ImageJ.

### Superresolution Imaging and 3D-Reconstruction of iPNs.

The iPNs were seeded on 8-well plates (Ibidi) at a density of 15,000 cells per well and incubated uninfected or infected with HEVcc for 5 d. Before imaging, cells were fixed and stained according to the previously stated protocol (see *Immunofluorescence staining*). For the morphological analysis and reconstruction, fluorescence microscopy was performed using a Zeiss Elyra PS.1 (Zeiss Elyra PS.1 LSM880, Carl Zeiss Microscopy GmbH, Germany) microscope and a 63× oil immersion objective (Plan-Apochromat 63×/1.4 Oil DIC, Carl Zeiss Microscopy GmbH, Germany). Structural illumination microscopy (SIM) was used to generate superresolution images from five phases and three rotations of the SIM grid. ZEN Black 2.3 was employed to process raw confocal SIM images. Imaris 9.8.0 (Oxford Instruments, UK) surface function was used for the reconstruction of the nucleus and the cell surface.

### Neurite Length Measurement.

To determine the length of neurites from HEVcc-inoculated iPNs and mock-inoculated iPNs, cells were seeded on 8-well plates (Ibidi) at a density of 15,000 cells per well. iPNs were either left uninfected or were inoculated with HEVcc. After 24 h, the inoculum of HEV-inoculated and mock-inoculated neurons was changed completely. Thereafter, half of the medium per well was changed every 2 d, ensuring minimal handling stress. Five days postinoculation, iPNs were fixed and immunofluorescence staining performed with the primary antibodies for β-III-tubulin and ORF2-encoded capsid protein. Images were acquired using the Keyence BZX800 microscope with 40× objective. Neurite length was measured by using the plugin NeuronJ (49) of the image analysis software ImageJ. Frequency distribution was calculated with GraphPad Prism 10.0.2.

### Transcriptome Analyses of Differentiation and HEV Infection of iPNs.

For transcriptomic analysis, primary neuronal cells were infected. Five days postinfection., RNA was isolated using the NucleoSpin RNA kit (#740955, Macherey-Nagel) according to the manufacturer’s instructions. Sequencing libraries were prepared using the NEBNext Ultra II Directional RNA Library Prep Kit and sequencing was conducted on the Illumina NovaSeq 6000 platform in 50-mer in paired end mode. All raw and processed files can be accessed online at https://www.ncbi.nlm.nih.gov/geo/, using the GEO accession code GSE275473 for RNA-Seq data related to primary neurons and GSE274780 for data related to primary human hepatocytes. Quality control, mapping against the human genome (Hg38) or HEV genome (Kernow-C1 p6 strain), and statistical analysis for gene expression were conducted in CLC Genomics Workbench 22.0. Gene expression was calculated for individual transcripts as reads per kilobase per million bases mapped (RPKM). Significant differentially regulated genes (DREGs) meeting the criteria of fold change (FC) > 1.5 or FC < -1.5, false discovery rate (FDR) < 0.05, and mean group TPM => 2. Genes identified in previous studies were employed to achieve a more detailed characterization of primary neuron cell types during the differentiation process (50, 51). IRGs were cross-referenced with the “Orthologous 9/24 Clusters of ISGs” database curated by the Centre for Virus Research (CVR), University of Glasgow, using specific criteria (log_2_FC > 0 and FDR < 0.05). The database is publicly available at http://isg.data.cvr.ac.uk (29). GO enrichment analyses for biological processes were performed by using the *Homo sapiens* EBI GO Annotation Database. Gene identifiers for DEGs (FC > 1.5 and max group mean > 2) were used as input for identification of significantly enriched GO categories. Single-cell RNA sequencing data were retrieved from public repositories using the CuratedAtlasQueryR library. Data visualization was done in the statistical programming language R with in-house scripts using the libraries tidyverse, tidytSingleCellExperiment, Seurat ggplot2, GO-plot, ComplexHeatmap, and venn.

### Software.

Quantification of the number of infected neurons was performed by an in-house Cellprofiler pipeline (52). This pipeline first detected the nuclei in the image and then distinguished whether a cell was differentiated based on the β-III-tubulin intensity around the nucleus. Additionally, the ORF2 intensity was measured for each cell to determine whether it was infected.

## Supplementary Material

Appendix 01 (PDF)

Movie S1. **The ORF2 encoded capsid protein of HEV is around the nucleus and in neurites** Confocal microscope images of HEV-infected neurons were acquired. A 3D reconstruction of the surfaces was performed using the analysis tool Imaris 9.8.0. Cells were stained with DAPI (blue), with an antibody against ORF2-encoded capsid protein (green) and with β-III-tubulin (red).

## Data Availability

Some study data are available, including RNA-Seq data related to primary neurons and primary human hepatocytes, which have been deposited in the GEO database under accession numbers GSE275473 and GSE274780. Previously published data were utilized for this work, where Interferon-Regulated Genes (IRGs) were cross-referenced with the “Orthologous 9/24 Clusters of ISGs” database. This database is curated by the CVR at the University of Glasgow, using specific criteria of log2FC > 0 and FDR < 0.05. The database is publicly accessible at http://isg.data.cvr.ac.uk (29). All other data are included in the manuscript and/or supporting information.
