# Supplementary material for: Modeling extrahepatic hepatitis E virus infection in induced human primary neurons
Source: Proc Natl Acad Sci U S A. 2024 Nov 15;121(47):e2411434121. doi: 10.1073/pnas.2411434121 (PMC11588080; doi:10.1073/pnas.2411434121)
Supplement: Supplementary file 1 — Appendix 01 (PDF) [file pnas.2411434121.sapp.pdf]

PNAS

Supporting Information for

Modeling extrahepatic hepatitis E virus infection in induced human primary neurons

Michelle Jagst[1,2], André Gömer[1], Sanja Augustyniak[3], Mara Klöhn[1], Adriana Rehm[3], Rainer G. Ulrich[4,5], Verian Bader[6], Konstanze F. Winklhofer[6], Yannick Brüggemann[1], Ralf Gold[3], Barbara Gisevius[3], Daniel Todt[1,7], Eike Steinmann[1,8#]

[1]Ruhr University Bochum, Institute for Hygiene and Microbiology, Department for Molecular and Medical Virology, Bochum, Germany

[2]University of Veterinary Medicine Hannover, Institute of Virology, Hannover, Germany

[3]Ruhr University Bochum, St. Josef Hospital, Department of Neurology, Bochum, Germany

[4]Friedrich-Loeffler-Institut, Institute of Novel and Emerging Infectious Diseases, Greifswald-Insel Riems, Germany

[5]German Centre for Infection Research, Partner site Hamburg-Lübeck-Borstel-Riems, Greifswald-Insel Riems, Germany.

[6]Ruhr University Bochum, Institute of Biochemistry and Pathobiochemistry, Department of Molecular Cell Biology, Bochum, Germany

[7]European Virus Bioinformatics Center (EVBC), Jena, Germany

[8]Department of Molecular and Medical Virology, Ruhr University Bochum, Bochum, Germany; German Centre for Infection Research (DZIF), External Partner Site, Bochum, Germany.

[#]**Corresponding author**:

Prof. Dr. Eike Steinmann

Department of Molecular and Medical Virology

Ruhr University Bochum, Universitätsstr. 150, 44801 Bochum, Germany

Phone: +49 234 32 23189, Fax: +49 234 32 14352

Email: eike.steinmann@rub.de

This PDF file includes:

Figures S1 to S8
Tables S1 to S2
Legend for Movie S1
SI References

Other supporting materials for this manuscript include the following:

Movies S1

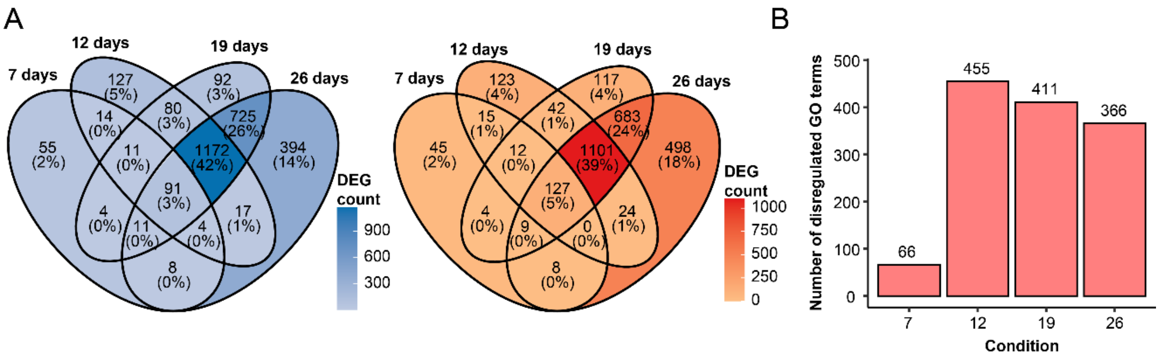

Fig. S1. **Regulation of genes during the neuron differentiation process**. **(A)** Venn diagram of significant down-regulated differentially expressed genes (DEGs, blue) and up-regulated DEGs (red) at different stages of differentiation compared to the earliest time point of 4 days. **(B)** Overview of the number of significant deregulated Gene ontology (GO) terms during the differentiation process.

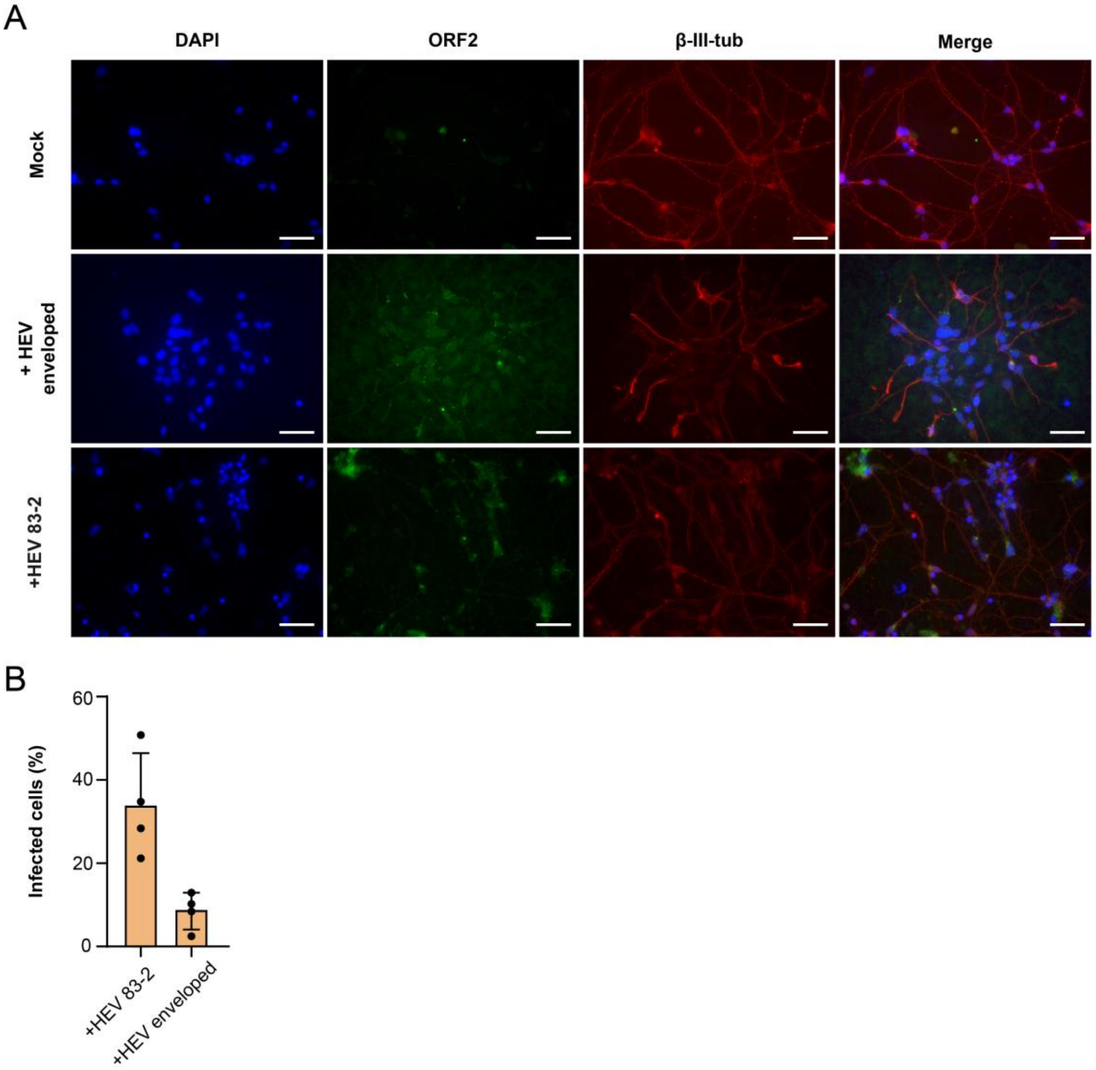

Fig. S2. Infection of human induced primary neurons with quasi-enveloped HEVcc Kernow-C1 p6 strain and wild boar HEV-3 strain 83-2. (A) Immunofluorescence staining was performed for uninfected (Mock) and infected induced primary neurons (iPNs) with quasi-enveloped Kernow-C1 p6 strain and the wild boar HEV-3 strain 83-2. 4′,6-diamidino-2-phenylindole (DAPI) was used to stain the nuclei (blue), a polyclonal rabbit anti-HEV antiserum to stain the ORF2-encoded capsid protein (green) and an anti-β-III-tubulin (β-III-Tub) antibody to stain the neuronal cytoskeleton. Scalebars represent 50 µm. (B) The susceptibility of neurons to both HEV strains was quantified by measuring the ORF2 signal using CellProfiler.

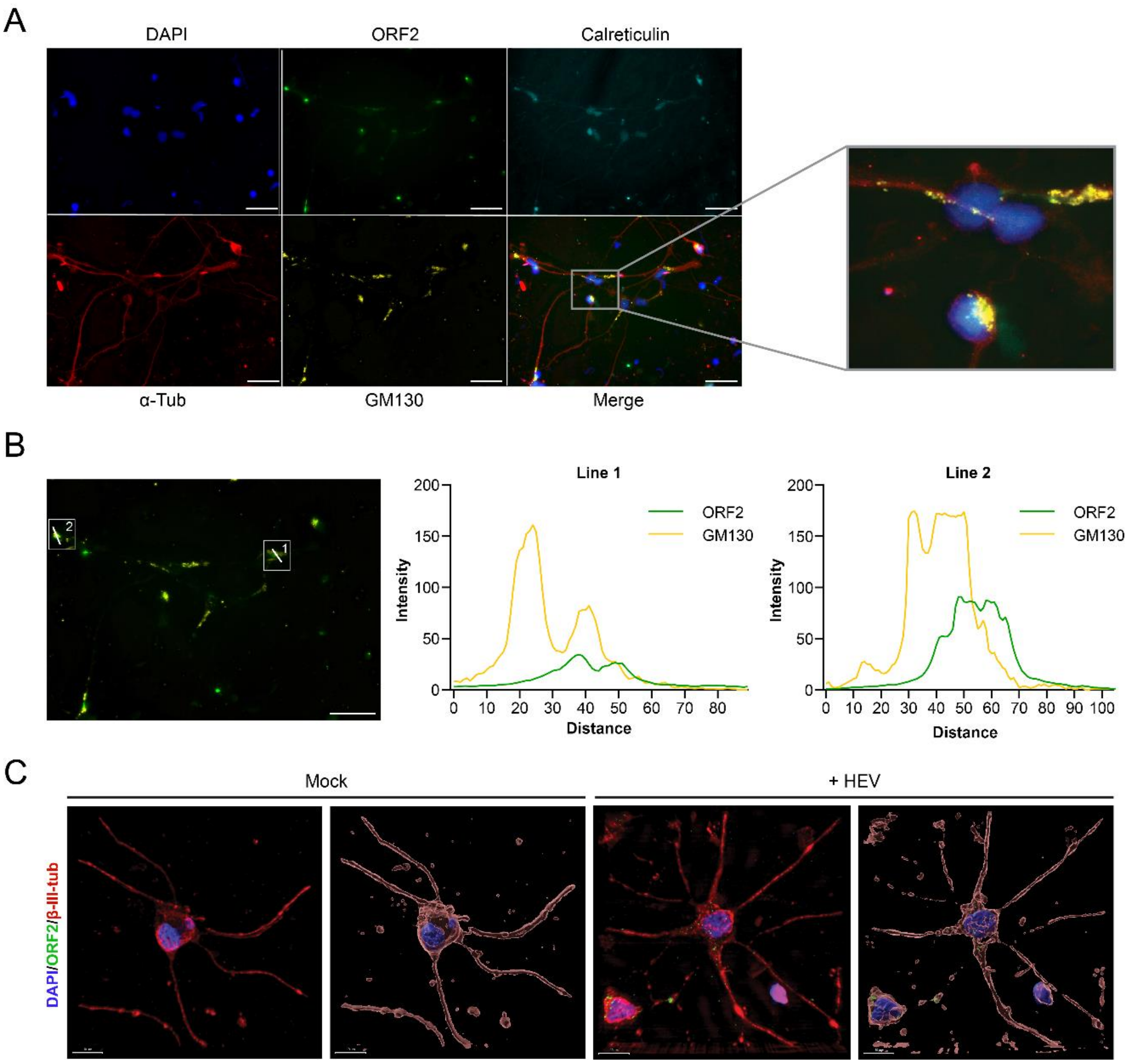

Fig. S3. The ORF2 encoded capsid protein of HEV is colocalized with the Golgi apparatus. (A) Virus localization was accessed by iterative indirect immunofluorescence imaging using multiple compartment markers. In different rounds of staining, antibodies of ORF2 (green), α-tubulin (α-Tub, red), GM130 (yellow) and calreticulin (cyan) were used to stain the HEV capsid protein, cytoskeleton, Golgi apparatus and the endoplasmic reticulum, respectively. The nucleus was stained by DAPI. White scalebar represents 50 µm **(B)** Line graphs show the fluorescence intensities of ORF2 capsid protein (green) and Golgi apparatus (GM130, yellow) measured across the regions of interest indicated by the white lines in the immunofluorescence image. White scalebar represents 50 µm. **(C)** Confocal microscope images of uninfected iPNs (Mock) and HEV-infected neurons were acquired. A 3D reconstruction of the surfaces was performed using the analysis tool Imaris 9.8.0 and a video was also generated. Cells were stained with DAPI (blue), with an antibody against ORF2-encoded capsid protein (green) and with β-III-tubulin (red). White scalebar represents 10 µm.

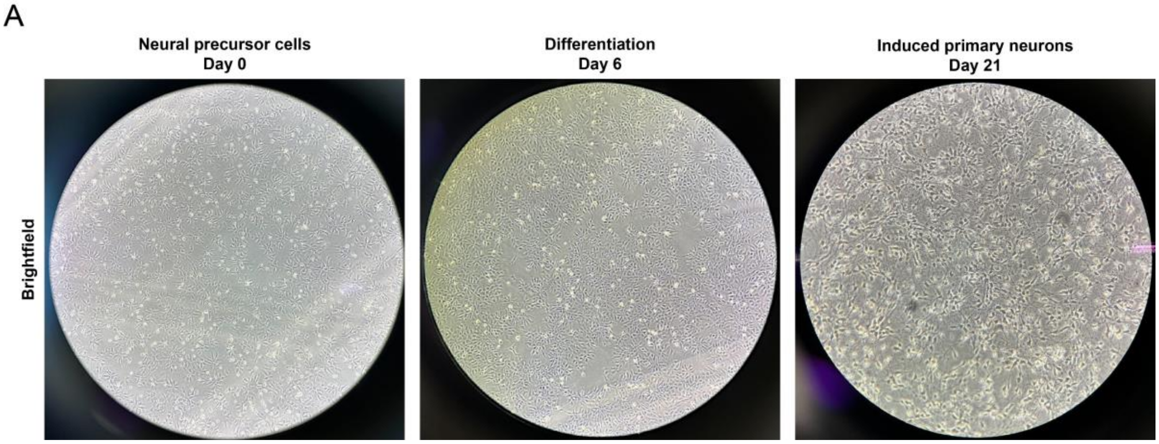

Fig. S4. Neurite development during differentiation into induced primary neurons. Brightfield images were taken at three differentiation timepoints: neural precursor cells (day 0), cells during differentiation (day 6), and induced primary neurons after 21 days of differentiation.

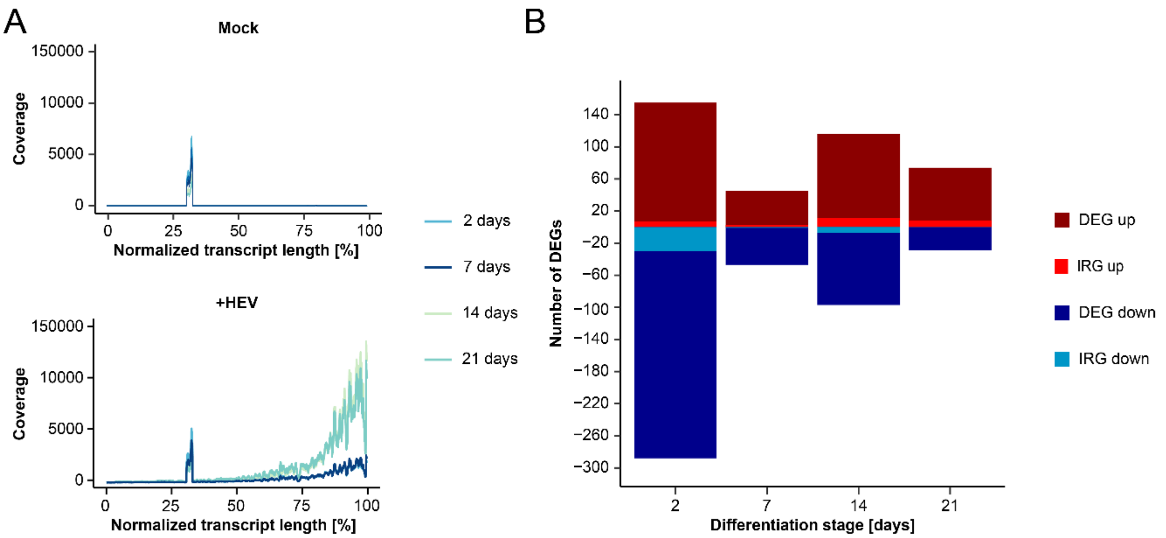

Fig. S5. General response of primary neurons to HEV infection. (A) Normalized coverage of mapped reads along the HEV genome in uninfected (Mock) and infected neurons (+HEV, five days post-infection) at the timepoints of 2, 7, 14 and 21 days of differentiation. **(B)** The number of differentially expressed genes (DEGs) by HEV infection compared to uninfected cells is shown over the course of differentiation. An online single-cell database of interferon (IFN)-regulated genes (IRGs) (http://isg.data.cvr.ac.uk/) was utilized, filtered for genes significantly up- or downregulated (FDR ≤ 0.05 and $\log_2$ fold change of 2 or -2) in humans, and were compared to the identified DEGs. Lighter colors indicate the number of IRGs that were significantly upregulated (red) or downregulated (blue).

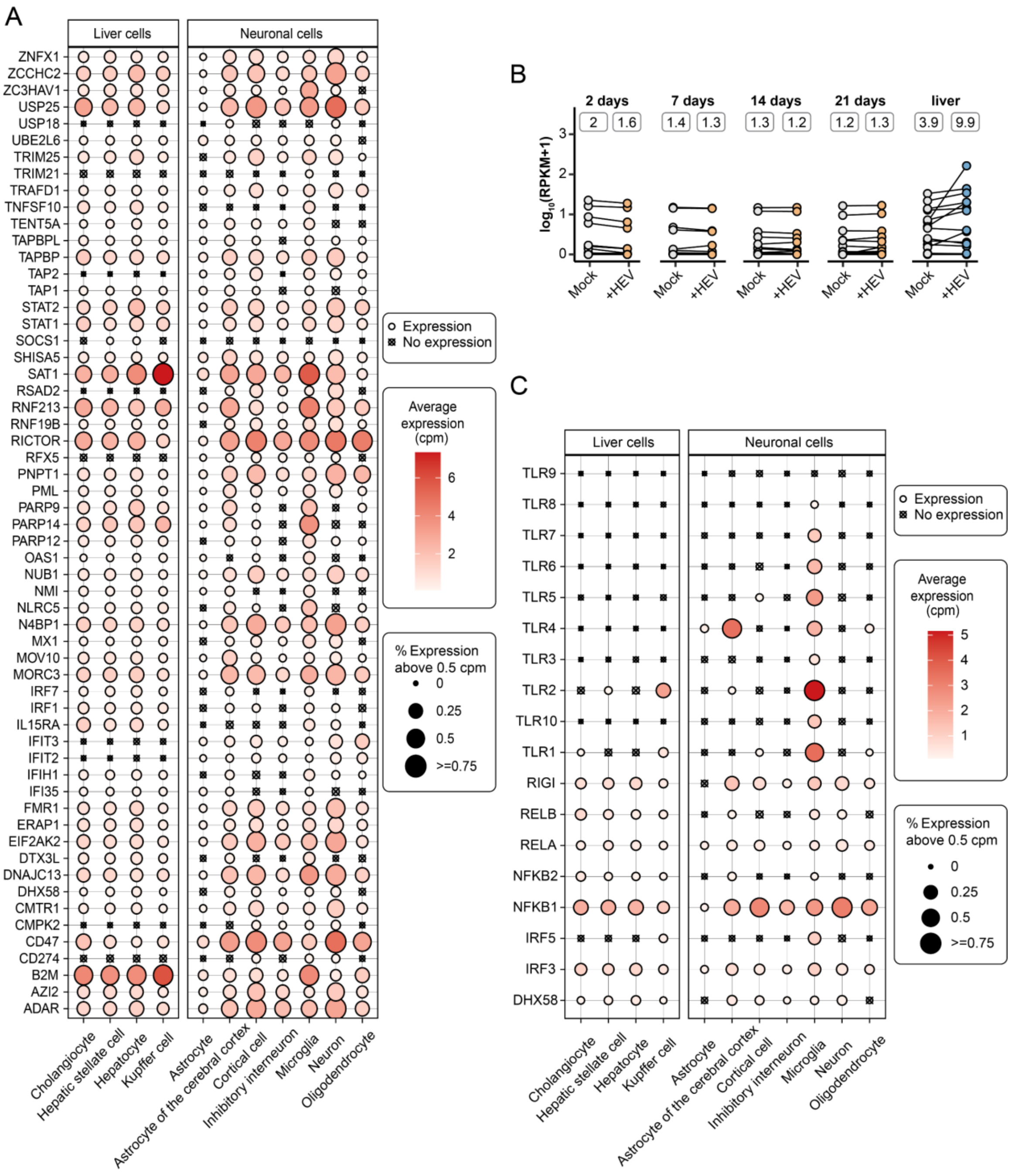

Fig. S6. Pathogen sensing in primary neurons and interferon-stimulated gene (ISG) expression across nervous system cell types. (A) Expression of ISGs in neuronal and liver cells in publicly available single-cell databases. **(B)** Overview and comparative analysis of the expression levels of pathogen-sensing genes measured in RPKM values, in both uninfected (Mock) and HEV-infected (+HEV) induced primary neurons and primary human hepatocytes (liver). The values above displayed represent the mean RPKM expression levels of all genes under each respective condition. **(C)** Expression of sensing genes in neuronal and liver cells in publicly

available single-cell databases. cpm: counts per million. RPKM: Reads per kilobase of transcript per million mapped reads.

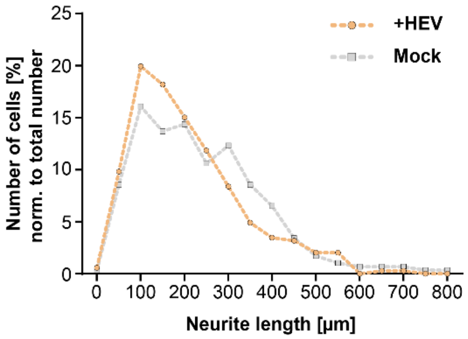

Fig. S7. Shift in neurite length after HEV inoculation. The neurite length of mock-inoculated (Mock) and HEV-inoculated (+HEV) iPNs (21-day-old cells) was examined through microscopic analysis five days post-inoculation. The frequency distribution is depicted as the percentage of cells (normalized to the total cell number) with a given neurite length.

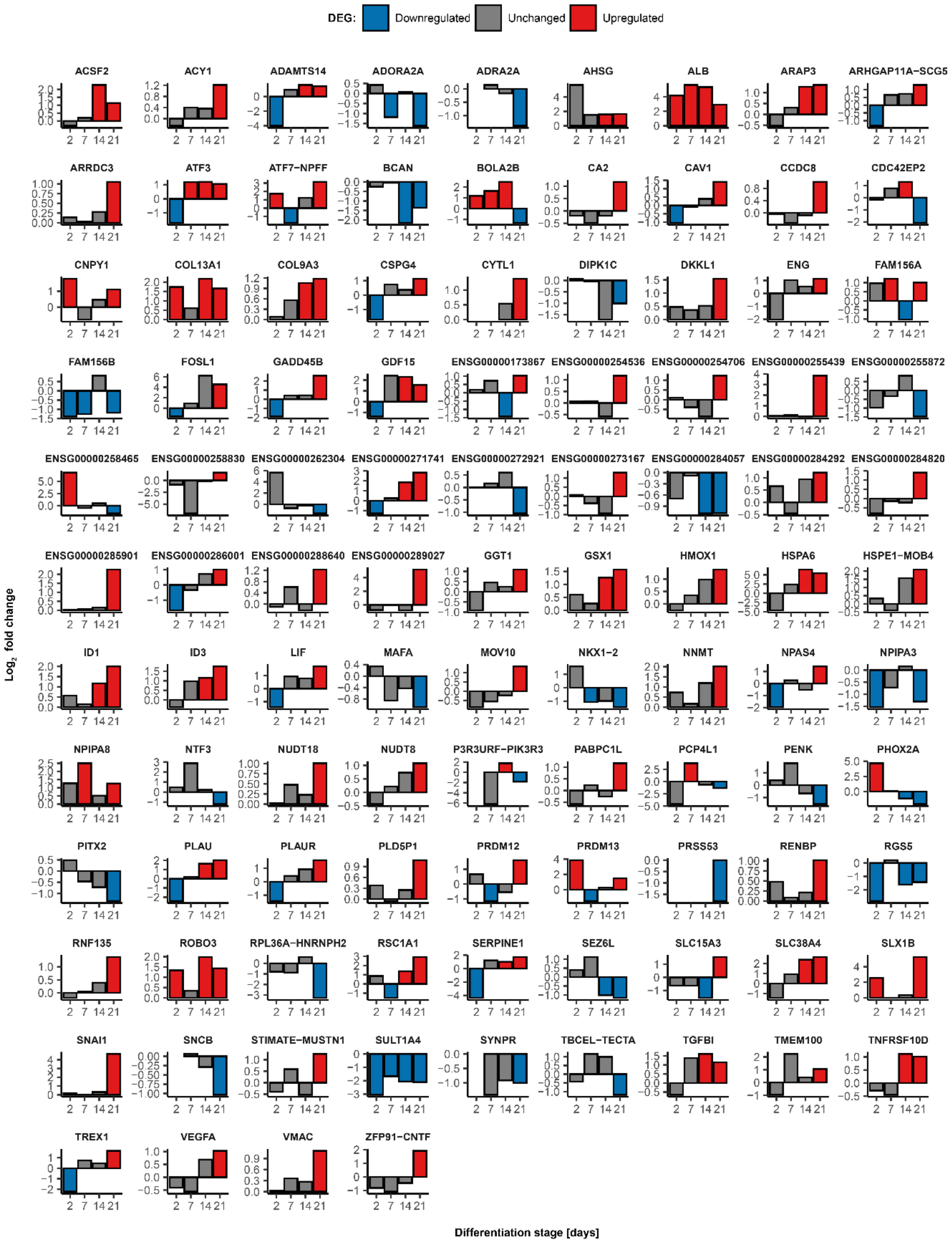
DEG: Downregulated Unchanged Upregulated
ACSF2 ACY1 ADAMTS14 ADORA2A ADRA2A AHSG ALB ARAP3 ARHGAP11A−SCG5
ARRDC3 ATF3 ATF7−NPFF BCAN BOLA2B CA2 CAV1 CCDC8 CDC42EP2
CNPY1 COL13A1 COL9A3 CSPG4 CYTL1 DIPK1C DKKL1 ENG FAM156A
FAM156B FOSL1 GADD45B GDF15 ENSG00000173867 ENSG00000254536 ENSG00000254706 ENSG00000255439 ENSG00000255872
ENSG00000258465 ENSG00000258830 ENSG00000262304 ENSG00000271741 ENSG00000272921 ENSG00000273167 ENSG00000284057 ENSG00000284292 ENSG00000284820
ENSG00000285901 ENSG00000286001 ENSG00000288640 ENSG00000289027 GGT1 GSX1 HMOX1 HSPA6 HSPE1−MOB4
ID1 ID3 LIF MAFA MOV10 NKX1−2 NNMT NPAS4 NPIPA3
NPIPA8 NTF3 NUDT18 NUDT8 P3R3URF−PIK3R3 PABPC1L PCP4L1 PENK PHOX2A
PITX2 PLAU PLAUR PLD5P1 PRDM12 PRDM13 PRSS53 RENBP RGS5
RNF135 ROBO3 RPL36A−HNRNPH2 RSC1A1 SERPINE1 SEZ6L SLC15A3 SLC38A4 SLX1B
SNAI1 SNCB STIMATE−MUSTN1 SULT1A4 SYNPR TBCEL−TECTA TGFBI TMEM100 TNFRSF10D
TREX1 VEGFA VMAC ZFP91−CNTF
Log2 fold change
Differentiation stage [days]

Fig. S8: Differential regulation of genes in 21-day-old neurons relative to various stages of differentiation. The genes presented display either upregulation or downregulation in 21-day-old primary neurons following HEV infection. These differentially regulated genes (DEGs) are depicted here in comparison with their expression patterns in 2, 7, and 14-day-old cells.

Table S1. Association between upregulated human genes at 21 days post-HEV infection and neurite length. Related genes include only genes of the same family. / indicating no correlation with neurite outgrowth.

Gene name (explanation)	Involved in neurite outgrowth regulation	Reported potential function of the encoded protein	Refer-ences	Log_2 fold change	False discovery rate (FDR)	Mock: Reads per kilobase of transcripts per million mapped reads	+HEV: Reads per kilobase of transcripts per million mapped reads
MOV10 (MOV10 RISC complex RNA helicase)	yes	Knockout of *MOV10* gene in murine neuroblastoma (Neuro2a) cells lead to reduced neurite length, which could be rescued upon re-induction of *MOV10* gene	(1, 2)	1.340118	4.392000e-09	0.58869067	1.49156581
ATF3 (Activating transcription factor 3)	yes	ATF3 promotes neurite outgrowth in rat dorsal root ganglion neurons	(3, 4)	1.055239	2.749000e-06	1.73958633	3.61697248
TREX1 (Three prime repair exonuclease 1)	yes	A *TREX1* mutant (V91M) lead to a significant reduction in axonal outgrowth in mouse primary cortical neurons compared to the wildtype cells	(5)	1.654444	2.098385e-02	0.2436382	0.7727430
TGFBI (Transforming growth factor beta induced)	yes	TGFB increased neurite outgrowth in scratched primary midbrain	(6–9)	1.148161	7.208000e-14	4.5132269	10.0050609

		cultures of dopaminergic cells (Wistar rats); TGFBI rescued neurite outgrowth in inhibitory environment via canonical ALK5/SMAD3 signaling in mouse N1E-115 neuron-like cells; TGF-/31 has a capability of promoting axonal regeneration in rat hippocampal neurons					
ARAP3 (ArfGAP with RhoGAP domain, ankyrin repeat and PH domain 3)	yes	Neurites outgrowth of rat pheochromocytoma (PC12) cells was affected by *ARAP3* under NGF or bFGF treatment	(10, 11)	1.355979	2.495000e-13	1.078767	2.762672
VEGFA (Vascular endothelial growth factor A)	yes	VEGF enhanced axonal/neurite outgrowth of mouse dorsal root and superior cervical ganglia, rat retinal ganglion cells and rat cerebral cortical neurons	(12–16)	1.027099	2.351000e-06	1.310088	2.671190
SERPINE1 (Serpin family E member 1)	yes	PAI-1 (plasminogen activator inhibitor-1, Serpine1), an endogenous inhibitor of tissue plasminogen activator (tPA), led to a decrease neurite length	(17, 18)	1.710986	1.403000e-09	0.54960125	1.80225088

		of rat primary neuronal progenitor cells					
CAV1 (Caveolin 1)	yes	Caveolin 1 was required for axonal outgrowth of motor neurons (from *Xenopus*); synapsin-Calveolin-1 enhanced axonal growth in differentiated NPCs derived from human iPSCs	(19, 20)	1.406676	3.374000e-04	0.3073646	0.8167771
ENG (Endoglin)	yes	Endoglin is a co-receptor of transforming growth factor β (TGF-β), thus it participates in TGF-β signaling pathway	(21)	1.130649	1.031000e-12	3.97019850	8.69529148
PLAU (Plasminogen activator, urokinase)	yes	Inhibition of uPA (another abbreviation for PLAU) gene expression reduced axonal length in Neuro2 cells and mouse dorsal root ganglia	(22, 23)	2.015380	1.463000e-10	0.44035605	1.78463341
FOSL1 (FOS like 1, AP-1 transcription factor subunit)	yes	FOSL1 siRNA injection in rats led to reduced axon growths in Schwann cells	(24)	4.547930	6.536000e-04	0.03115952	0.77415355
NPAS4 (Neuronal PAS domain protein 4)	yes	*NPAS4* gene knockdown in Neuro2a cells inhibited neurite outgrowth; In NPAS4 knockout hippocampal	(25)	1.423217	3.957000e-04	0.31114766	0.83648270

		neurons (mouse) neurite outgrowth was abolished					
NNMT (Nicotinamide N-methyltransferase)	yes	NNMT stable expression in SH-SY5Y cells reduced the neurite length compared to SH-SY5Y cells without NNMT, whereas the number of neurites and branches increased	(26)	2.016775	1.567549e-02	0.1095445	0.4487772
ROBO3 (Roundabout guidance receptor 3)	yes	The lack of ROBO3 in mouse led to a decreased axon outgrowth in spinal cord explants	(27)	1.433759	4.375000e-30	17.05178	46.07125
CSPG4 (Chondroitin sulfate proteoglycan 4)	yes	CSPG reduced neurite length in mouse cerebral cortical neurons and granule cells in a dose-dependent manner; led also in human-derived SH-SY5Y cells to reduction of neurite length; CSPG reduced neurite length in mouse cerebellar granule cells in a dose-dependent manner; NG2 (synonym for CSPG4) also inhibited neurite growth from embryonic rat	(28–32)	1.128576	3.951000e-06	0.40782702	0.89232606

		dorsal root ganglia neurons					
ACSF2 (Acyl-CoA synthetase family member 2)	yes	ACSF2-deficient Neuro2a cells showed significantly blunted neurite outgrowth in response to retinoic acid; Acyl-CoA synthetase 2 overexpression enhanced neurite outgrowth in PC12 cells	(33, 34)	1.136887	2.484000e-03	0.5538275	1.2198368
GADD45B (Growth arrest and DNA damage inducible beta)	yes	Mutated GADD45B mice showed impaired activity-dependent neurogenesis and dendritic development; GADD45G/p38 MAPK/CDC25B signaling pathway enhanced neurite outgrowth by promoting microtubule polymerization	(35–37)	2.575122	1.250000e-14	0.5593153	3.3446735
GDF15 (Growth differentiation factor 15)	yes	The neurites length of the primary culture of rat retinal ganglion cells were increased by GDF15 treatment	(38)	1.549360	2.145562e-02	0.20693118	0.60944003
PLAUR (Plasminogen activator, urokinase receptor)	yes	Exogenous uPA (PLAU) increased neurite length in mouse Neuro2a cells	(23, 39)	1.597618	8.919000e-05	0.2644716	0.8027418

		most likely via the interaction with uPAR (PLAUR); blocking of uPAR stimulated neurite formation					
CCDC8<br>(Coiled-coil domain containing 8)	yes	CCDC8 increased the number of neurites per cell in rat PC12 cells	(40)	1.014808	3.547000e-07	1.96235660	3.96682179
LIF<br>(LIF interleukin 6 family cytokine)	yes	LIF promoted neurite outgrowth of rat mammalian auditory neurons in vitro; LIF promoted neurite growth of P12-E2 cells	(41, 42)	1.708004	1.181000e-03	0.14429893	0.47372902
HSPA6<br>(Heat shock protein family A (Hsp70) member 6)	Related genes	HSP70 alone and especially in combination with HSP40 increased neurite outgrowth in Neuro2a cells;<br>Hsp27 is involved in GDNF-induced neurite outgrowth in rat PC12-GFRR1-RET cells;<br>Neurite outgrowth was mediated by the heat shock protein HSP90a	(43–45)	5.420693	4.269000e-49	0.23718946	10.2284998 4
CNPY1<br>(Canopy FGF signaling regulator 1)	Related genes	CNPY2 (MSAP) increased the number of neurites on mouse N2-A and rat PC12 cells	(46)	1.105491	1.345000e-06	0.9920355	2.1358922

TNFRSF10D (TNF receptor superfamily member 10d)	Related genes	Tumor necrosis factor (TNF) decreased the total neurite length per cell, the axonal length and the number of branch points of primary mice hippocampal neurons cocultured with astrocyte-enriched glial cells	(47)	1.009861	6.689000e-05	0.9730365	1.9607246
CA2 (Carbonic anhydrase 2)	Related genes	CA8 induced neurite outgrowth in Neuro-2a cells but not in human SK-N-SH cells	(48)	1.173081	1.356000e-05	1.55652100	3.51307191
PRDM12 (PR/SET domain 12)	Related genes	Disruption of the PRDM14 gene resulted in axon growth defects in zebrafish	(49)	1.605972	2.794000e-13	1.303463	3.971309
PLD5P1 (Phospholipase D family member 5 pseudogene 1)	Related genes	Phospholipase D1 inhibited neurite outgrowth of rat neural stem cells via increase of synapsin 1 expression; Overexpression of PLD1 resulted in neurons to increase tissue plasminogen activator (tPA) release and therefore to tPA-dependent neurite extension	(50, 51)	1.077125	2.514724e-02	0.2453015	0.5186789

ADAMTS14 (ADAM metallopeptidase with thrombospondin type 1 motif 14)	Related genes	ADAMTS4 and ADAMTS5 led to an increase in neurite length in rat primary neurons	(52)	1.414671	3.603000e-06	0.3116316	0.8320400
SLC15A3 (Solute carrier family 15 member 3)	Related genes	SLC25A12 expression was associated with dendrites outgrowth in mouse embryonic cortical neurons	(53)	1.547206	7.039000e-03	0.1887240	0.5542340
SLC38A4 (Solute carrier family 38 member 4)	Related genes	SLC25A12 expression was associated with dendrites outgrowth in mouse embryonic cortical neurons	(53)	2.649440	2.497000e-05	0.07092644	0.44991230
RNF135 (Ring finger protein 135)	Related genes	Molluscan RING-finger protein L-TRIM is essential for neuronal outgrowth	(54)	1.374997	4.669022e-02	0.1963424	0.5120273
VMAC (Vimentin type intermediate filament associated coiled-coil protein)	Related genes	The length of astrocytic processes after entorhinal cortex lesion was shorter in GFAP-/-Vim-/- than wildtype mice (Vim=Vimentin)	(55)	1.107776	3.657000e-03	0.6299788	1.3598131
DKKL1 (Dickkopf like acrosomal protein 1)	Related genes	Dickkopf-1 protein (Dkk1) induced neurite outgrowth in TC-32 cells	(56)	1.540152	2.998727e-02	0.2884431	0.8444769
SNAI1	Related genes	SNAIL transcription factor in prostate cancer	(57)	4.665358	6.446000e-06	0.05172846	1.36721883

(Snail family transcriptional repressor 1)		cells promoted neurite outgrowth					
RSC1A1 (regulator of solute carriers 1)	/	/	/	2.927757	3.623000e-04	0.07599916	0.58922164
ID3 (Inhibitor Of DNA Binding 3)	/	/	/	1.748242	3.465000e-30	11.0787844 4	37.2322951 4
ACY1 (Aminoacylase 1)	/	/	/	1.214575	5.344000e-03	0.4597700	1.0693794
AHSG (Alpha 2-HS glycoprotein)	/	/	/	1.638499	6.277000e-04	0.4287744	1.3402270
CYTL1 (Cytokine like 1)	/	/	/	1.380700	1.976895e-02	0.5647939	1.4770756
ALB (albumin)	/	/	/	2.926996	1.716000e-22	0.45646804	3.48247791
ARRDC3 (Arrestin domain containing 3)	/	/	/	1.051950	6.683000e-14	8.266954	17.142819
PRDM13 (PR/SET domain 13)	/	/	/	1.489413	5.248000e-03	0.19102079	0.53853983
NUDT18 (Nudix hydrolase 18)	/	/	/	1.008633	7.463000e-03	0.8883395	1.7897523
COL13A1 (Collagen type XIII alpha 1 chain)	/	/	/	1.657751	1.174000e-11	0.6850415	2.1640083
NUDT8 (Nudix hydrolase 8)	/	/	/	1.076710	3.940508e-02	0.8883395	1.7897523

GSX1<br>(GS homeobox 1)	/	/	/	1.575707	5.544000e-03	0.2902809	0.8695302
NPIPA8<br>(Nuclear pore complex interacting protein family member A8)	/	/	/	1.261234	4.423000e-03	0.59556003	1.43100559
SLX1B<br>(SLX1 homolog B, structure-specific endonuclease subunit)	/	/	/	5.257244	3.554000e-20	0.1814754	7.0508826
TMEM100<br>(Transmembrane protein 100)	/	/	/	1.055878	2.705273e-02	0.37648448	0.78435276
ID1<br>(Inhibitor Of DNA Binding 1)	/	/	/	2.002127	7.104000e-13	1.28754784	5.16763625
PABPC1L<br>(Poly(A) binding protein cytoplasmic 1 like)	/	/	/	1.165967	2.460000e-04	0.60324994	1.35526644
COL9A3<br>(Collagen type IX alpha 3 chain)	/	/	/	1.177167	3.118000e-03	0.4791457	1.0854985
GGT1<br>(Gamma-glutamyltransferase 1)	/	/	/	1.093269	4.897000e-03	0.34972246	0.74733914
HMOX1<br>(Heme oxygenase 1)	/	/	/	1.390076	7.152000e-06	0.8629264	2.2650443
FAM156A	/	/	/	1.007986	1.842000e-10	2.654675	5.340031

(Family with sequence similarity 156 member A)							
RENBP (Renin binding protein)	/	/	/	1.017142	3.818000e-03	1.112195	2.253698
gene:*ENSG00000271741*	/	/	/	2.823195	1.416000e-05	0.0853795	0.6117999
gene:*ENSG00000254706*	/	/	/	1.233511	1.727228e-02	0.6527534	1.5394736
HSPE1-MOB4 Readthrough	/	/	/	2.099540	1.567000e-03	0.3633585	1.5715336
gene:*ENSG00000284820*	/	/	/	1.405860	6.634000e-05	0.2522317	0.6696288
STIMATE-MUSTN1 Readthrough	/	/	/	1.252719	4.557356e-02	0.3728846	0.8921909
gene:*ENSG00000286001*	/	/	/	1.012625	2.275000e-03	0.5374444	1.0855183
gene:*ENSG00000289027*	/	/	/	5.189919	2.232000e-12	0.02737338	1.02447209
gene:*ENSG00000284292*	/	/	/	1.213351	2.323063e-02	0.2717099	0.6319702
gene:*ENSG00000288640*	/	/	/	1.230503	4.934000e-09	1.4441618	3.3907027
gene:*ENSG00000254536*	/	/	/	1.179328	2.286894e-02	0.3346430	0.7600015
ZFP91-CNTF Readthrough	/	/	/	1.918956	8.172000e-04	0.1824514	0.6944137
gene:*ENSG00000285901*	/	/	/	2.267598	7.375000e-15	0.3936985	1.9001755

ATF7-NPFF Readthrough	/	/	/	3.141335	1.104214e-02	0.05481481	0.50224828
gene:*ENSG00000258830*	/	/	/	1.653482	2.940749e-02	0.15348320	0.48679998
gene:*ENSG00000273167*	/	/	/	1.298390	8.305000e-03	0.1909486	0.4710442
ARHGAP11A-SCG5 Readthrough	/	/	/	1.304814	6.584000e-04	0.3203187	0.7929361
gene:*ENSG00000173867*	/	/	/	1.029718	4.505890e-02	0.2787915	0.5704761
gene:*ENSG00000255439*	/	/	/	3.833119	9.724000e-09	0.07577064	1.09860765

Table S2. Association between downregulated human genes at 21 days post-HEV infection and neurite length. Related genes include only genes of the same family. / indicating no correlation with neurite outgrowth.

Gene name (explanation)	Involved in neurite outgrowth regulation	Reported potential function of the encoded protein	References	Log_2 fold change	False discovery rate (FDR)	Mock: Reads per kilobase of transcripts per million mapped reads	+HEV: Reads per kilobase of transcripts per million mapped reads
BCAN (Brevican)	yes	Brevican inhibits neurite outgrowth from rat cerebellar granule neurons; Brevican is an inhibitor of neurite outgrowth in the proteoglycan-enriched myelin fraction in cerebellar granule cells	(58, 59)	-1.358337	1.465000e-21	13.3752915	5.2167392
PCP4L1 (Purkinje cell protein 4 like 1)	yes	PCP4 enhance neurite outgrowth, while a knockdown reduces neurite outgrowth in PC12 cells	(60)	-1.344553	1.421000e-04	2.53007119	0.99478298
RGS5 (Regulator of G protein signaling 5)	yes	RGS5 leads to an inhibitory effect of neurite outgrowth by sonic hedgehog in mouse primary cortical neurons; RGS5 switched astrocytes from neuroprotective to	(61, 62)	-1.426282	8.729000e-10	1.3988044	0.5201488

	yes	pro-inflammatory property via binding to the receptor TNFR2					
PITX2 (Paired like homeodomain 2)	yes	Loss of PITX2 disrupts axonal growth in the dorsal midbrain in mouse	(63)	-1.336801	3.957000e-04	0.60078189	0.23744417
PENK (Proenkephalin)	yes	Proenkephalin produces the peptide [Met]enkephalin and [Let]enkephalin via proteolytic cleavage; [Met]enkephalin and [Leu]enkephalin both has a neurite growth-promoting effect in rat dorsal root ganglia	(64)	-1.529499	1.205000e-10	2.83594972	0.98163152
CDC42EP2 (CDC42 effector protein 2)	yes	bind to, and negatively regulate the function of CDC42; CDC42 stimulates neurite outgrowth in primary chick spinal cord neurons ; Cdc42 plays a l role in neurite outgrowth of PC12 cells and cerebellar granule neurons	(65, 66)	-2.061407	8.766000e-05	0.9645701	0.2298073
NTF3 (Neurotrophin 3)	yes	NTF3 promotes neurite outgrowth in rat hippocampal cells and sensory neurons	(67–69)	-1.199443	3.067719e-02	1.17344583	0.50953183

ADORA2A (Adenosine A2a receptor)	yes	An ADORA2A antagonist rescued the decreased neurite length in rat primary cortical neurons from an attention deficit and hyperactivity disorder model; the suppression of ADORA2A seems to correlate with neurite outgrowth promoted by ultrasound stimulation in PC12 cells	(70, 71)	-1.614159	8.454000e-06	1.0091193	0.3289512
SNCB (Synuclein beta)	Related genes	Alpha-Synuclein reversed the SPTBN1-induced neurite over-branching in SH-SY5Y cells; Beta-synuclein is discussed as a natural negative regulator of alpha-synuclein aggregation	(72, 73)	-1.033695	2.206000e-05	4.7782508	2.3330362
PHOX2A (Paired like homeobox 2A)	Related genes	PHOX2B knockdown reduced neurite length in human motor neurons and induced short spinal neurons in zebrafish	(74)	-2.067991	3.509352e-02	0.4672003	0.1097638
SEZ6L (Seizure related 6 homolog like)	Related genes	SEZ6 suppression leads to decreased neurite length in PC12 cells; SEZ6 family member are	(75, 76)	-1.166480	2.178000e-07	1.35654181	0.60410517

		known to affect neurite length (SEZ6 and SEZ6L2)					
SYNPR (Synaptoporin)	/	/	/	-1.002154	4.107603e-02	0.66902816	0.33344849
MAFA (MAF bZIP transcription factor A)	/	/	/	-1.084093	4.790000e-07	3.8772766	1.8283201
ADRA2A (Adrenoceptor alpha 2A)	/	/	/	-1.381024	2.780000e-08	1.8795963	0.7211471
NKX1-2 (NK1 homeobox 2)	/	/	/	-1.445285	4.276000e-08	2.00510214	0.73565542
NPIPA3 (Nuclear pore complex interacting protein family member A3)	/	/	/	-1.304036	2.042396e-02	0.7830245	0.3160825
SULT1A4 (Sulfotransferase family 1A member 4)	/	/	/	-2.107897	7.278000e-06	1.7434995	0.4025545
BOLA2B (BolA family member 2B)	/	/	/	-1.307911	1.149725e-02	1.4269165	0.5746954
PRSS53 (Serine protease 53)	/	/	/	-1.794549	2.217000e-07	1.8605236	0.5351388
DIPK1C (FAM69C)	/	/	/	-1.007293	2.264083e-02	0.76087239	0.37797824

(Divergent protein kinase domain 1C)							
FAM156B (TMEM29B) (Family with sequence similarity 156 member B)	/	/	/	-1.189186	9.333000e-14	6.426085	2.817976
P3R3URF-PIK3R3 Readthrough	/	/	/	-1.833480	1.452810e-02	0.7193576	0.2001381
gene:*ENSG0000025 8465*	/	/	/	-1.602100	1.054000e-05	1.09259278	0.35915470
gene:*ENSG0000025 5872*	/	/	/	-1.477783	3.862000e-03	0.7375282	0.2639103
gene:*ENSG0000028 4057*	/	/	/	-1.088332	1.605000e-07	3.8209871	1.7965551
TBCEL-TECTA Readthrough	/	/	/	-1.183985	4.005257e-02	1.0880918	0.4775082
gene:*ENSG0000027 2921*	/	/	/	-1.046384	3.162111e-02	2.515808	1.215900
gene:*ENSG0000026 2304*	/	/	/	-1.626821	2.406000e-05	0.66801872	0.21579793
RPL36A-HNRNPH2 Readthrough	/	/	/	-3.273787	1.058000e-03	1.776631	0.178886

Movie S1 (separate file): The ORF2 encoded capsid protein of HEV is around the nucleus and in neurites Confocal microscope images of HEV-infected neurons were acquired. A 3D reconstruction of the surfaces was performed using the analysis tool Imaris 9.8.0. Cells were stained with DAPI (blue), with an antibody against ORF2-encoded capsid protein (green) and with β-III-tubulin (red).

SI References

1. P. J. Kenny, *et al.*, The FMRP–MOV10 complex: a translational regulatory switch modulated by G-Quadruplexes. *Nucleic Acids Res.* **48**, 862–878 (2020).

2. G. Skariah, *et al.*, Mov10 suppresses retroelements and regulates neuronal development and function in the developing brain. *BMC Biol.* **15**, 54 (2017).

3. H. R. Katz, A. A. Arcese, O. Bloom, J. R. Morgan, Activating Transcription Factor 3 (ATF3) is a Highly Conserved Pro-regenerative Transcription Factor in the Vertebrate Nervous System. *Front. Cell Dev. Biol.* **10** (2022).

4. R. Seijffers, A. J. Allchorne, C. J. Woolf, The transcription factor ATF-3 promotes neurite outgrowth. *Mol. Cell. Neurosci.* **32**, 143–154 (2006).

5. D. Halder, *et al.*, TREX1 Deficiency Induces ER Stress-Mediated Neuronal Cell Death by Disrupting Ca2+ Homeostasis. *Mol. Neurobiol.* **59**, 1398–1418 (2022).

6. K. Abe, P. Chu, A. Ishihara, H. Saito, Transforming growth factor-β1 promotes re-elongation of injured axons of cultured rat hippocampal neurons. *Brain Res.* **723**, 206–209 (1996).

7. J. Kaiser, *et al.*, TGFβ1 Induces Axonal Outgrowth via ALK5/PKA/SMURF1-Mediated Degradation of RhoA and Stabilization of PAR6. *eNeuro* **7**, ENEURO.0104-20.2020 (2020).

8. J. Knöferle, *et al.*, TGF-β 1 enhances neurite outgrowth via regulation of proteasome function and EFABP. *Neurobiol. Dis.* **38**, 395–404 (2010).

9. S. Li, X. Gu, S. Yi, The Regulatory Effects of Transforming Growth Factor-β on Nerve Regeneration. *Cell Transplant.* **26**, 381–394 (2017).

10. C.-Y. Jeon, *et al.*, p190RhoGAP and Rap-dependent RhoGAP (ARAP3) inactivate RhoA in response to nerve growth factor leading to neurite outgrowth from PC12 cells. *Exp. Mol. Med.* **42**, 335–344 (2010).

11. C.-Y. Jeon, *et al.*, Neurite outgrowth from PC12 cells by basic fibroblast growth factor (bFGF) is mediated by RhoA inactivation through p190RhoGAP and ARAP3. *J. Cell. Physiol.* **224**, 786–794 (2010).

12. S. Böcker-Meffert, *et al.*, Erythropoietin and VEGF promote neural outgrowth from retinal explants in postnatal rats. *Invest. Ophthalmol. Vis. Sci.* **43**, 2021–2026 (2002).

13. K. Jin, X. O. Mao, D. A. Greenberg, Vascular endothelial growth factor stimulates neurite outgrowth from cerebral cortical neurons via Rho kinase signaling. *J. Neurobiol.* **66**, 236–242 (2006).

14. A. A. Khaibullina, J. M. Rosenstein, J. M. Krum, Vascular endothelial growth factor promotes neurite maturation in primary CNS neuronal cultures. *Brain Res. Dev. Brain Res.* **148**, 59–68 (2004).

15. J. M. Rosenstein, N. Mani, A. Khaibullina, J. M. Krum, Neurotrophic effects of vascular endothelial growth factor on organotypic cortical explants and primary cortical neurons. *J. Neurosci. Off. J. Soc. Neurosci.* **23**, 11036–11044 (2003).

16. M. Sondell, G. Lundborg, M. Kanje, Vascular endothelial growth factor has neurotrophic activity and stimulates axonal outgrowth, enhancing cell survival and Schwann cell proliferation in the peripheral nervous system. *J. Neurosci. Off. J. Soc. Neurosci.* **19**, 5731–5740 (1999).

17. S. H. Lee, *et al.*, tPA Regulates Neurite Outgrowth by Phosphorylation of LRP5/6 in Neural Progenitor Cells. *Mol. Neurobiol.* **49**, 199–215 (2014).

18. H. Xin, *et al.*, Increasing tPA activity in astrocytes induced by multipotent mesenchymal stromal cells facilitate neurite outgrowth after stroke in the mouse. *PloS One* **5**, e9027 (2010).

19. M. Breuer, H. Berger, A. Borchers, Caveolin 1 is required for axonal outgrowth of motor neurons and affects Xenopus neuromuscular development. *Sci. Rep.* **10**, 16446 (2020).

20. S. Wang, *et al.*, Caveolin-1 Phosphorylation Is Essential for Axonal Growth of Human Neurons Derived From iPSCs. *Front. Cell. Neurosci.* **13** (2019).

21. M. L. Mancini, *et al.*, Endoglin is required for myogenic differentiation potential of neural crest stem cells. *Dev. Biol.* **308**, 520–533 (2007).

22. M. A. Dent, Y. Sumi, R. J. Morris, P. J. Seeley, Urokinase-type plasminogen activator expression by neurons and oligodendrocytes during process outgrowth in developing rat brain. *Eur. J. Neurosci.* **5**, 633–647 (1993).

23. E. Semina, *et al.*, Urokinase and urokinase receptor participate in regulation of neuronal migration, axon growth and branching. *Eur. J. Cell Biol.* **95**, 295–310 (2016).

24. Q. Chen, L. Zhang, F. Zhang, S. Yi, FOSL1 modulates Schwann cell responses in the wound microenvironment and regulates peripheral nerve regeneration. *J. Biol. Chem.* **299** (2023).

25. J. Yun, *et al.*, Neuronal Per Arnt Sim (PAS) domain protein 4 (NPAS4) regulates neurite outgrowth and phosphorylation of synapsin I. *J. Biol. Chem.* **288**, 2655–2664 (2013).

26. M. G. Thomas, *et al.*, Nicotinamide N-methyltransferase expression in SH-SY5Y neuroblastoma and N27 mesencephalic neurones induces changes in cell morphology via ephrin-B2 and Akt signalling. *Cell Death Dis.* **4**, e669 (2013).

27. M. Barber, *et al.*, The Role of Robo3 in the Development of Cortical Interneurons. *Cereb. Cortex N. Y. NY* **19**, i22–i31 (2009).

28. C. L. Dou, J. M. Levine, Inhibition of neurite growth by the NG2 chondroitin sulfate proteoglycan. *J. Neurosci. Off. J. Soc. Neurosci.* **14**, 7616–7628 (1994).

29. D. L. Hynds, D. M. Snow, Neurite Outgrowth Inhibition by Chondroitin Sulfate Proteoglycan: Stalling/Stopping Exceeds Turning in Human Neuroblastoma Growth Cones. *Exp. Neurol.* **160**, 244–255 (1999).

30. J. Jin, *et al.*, Effect of chondroitin sulfate proteoglycans on neuronal cell adhesion, spreading and neurite growth in culture. *Neural Regen. Res.* **13**, 289–297 (2018).

31. G. Loers, *et al.*, Identification and characterization of synthetic chondroitin-4-sulfate binding peptides in neuronal functions. *Sci. Rep.* **9**, 1064 (2019).

32. Y. M. Ughrin, Z. J. Chen, J. M. Levine, Multiple Regions of the NG2 Proteoglycan Inhibit Neurite Growth and Induce Growth Cone Collapse. *J. Neurosci.* **23**, 175–186 (2003).

33. D. Maiguel, M. Morita, Z. Pei, Z. Jia, P. A. Watkins, ACSF2: A MEDIUM-CHAIN ACYL-CoA SYNTHETASE WITH A POTENTIAL ROLE IN NEURONAL DIFFERENTIATION. [Preprint] (2022). Available at: http://biorxiv.org/lookup/doi/10.1101/2022.03.28.486105 [Accessed 29 April 2024].

34. J. R. Marszalek, C. Kitidis, A. Dararutana, H. F. Lodish, Acyl-CoA synthetase 2 overexpression enhances fatty acid internalization and neurite outgrowth. *J. Biol. Chem.* **279**, 23882–23891 (2004).

35. Y. Kase, T. Sato, Y. Okano, H. Okano, The GADD45G/p38 MAPK/CDC25B signaling pathway enhances neurite outgrowth by promoting microtubule polymerization. *iScience* **25**, 104089 (2022).

36. D. K. Ma, *et al.*, Neuronal Activity–Induced Gadd45b Promotes Epigenetic DNA Demethylation and Adult Neurogenesis. *Science* **323**, 1074–1077 (2009).

37. E. Matsunaga, S. Nambu, M. Oka, A. Iriki, Comparative analysis of developmentally regulated expressions of *Gadd45a*, *Gadd45b*, and *Gadd45g* in the mouse and marmoset cerebral cortex. *Neuroscience* **284**, 566–580 (2015).

38. Y. Iwata, *et al.*, The effect of GDF15 on the neurite outgrowth in retinal ganglion cells. *Invest. Ophthalmol. Vis. Sci.* **60**, 624 (2019).

39. P. S. Klimovich, *et al.*, Urokinase receptor regulates nerve regeneration through its interaction with α5β1-integrin. *Biomed. Pharmacother.* **125**, 110008 (2020).

40. V. Laketa, J. C. Simpson, S. Bechtel, S. Wiemann, R. Pepperkok, High-Content Microscopy Identifies New Neurite Outgrowth Regulators. *Mol. Biol. Cell* **18**, 242–252 (2007).

41. L. N. Gillespie, G. M. Clark, P. F. Bartlett, P. L. Marzella, LIF is more potent than BDNF in promoting neurite outgrowth of mammalian auditory neurons in vitro. *Neuroreport* **12**, 275–279 (2001).

42. Y. Y. Wu, R. A. Bradshaw, Induction of Neurite Outgrowth by Interleukin-6 Is Accompanied by Activation of Stat3 Signaling Pathway in a Variant PC12 Cell (E2) Line*. *J. Biol. Chem.* **271**, 13023–13032 (1996).

43. Z. Hong, *et al.*, Phosphoproteome Study Reveals Hsp27 as a Novel Signaling Molecule Involved in GDNF-Induced Neurite Outgrowth. *J. Proteome Res.* **8**, 2768–2787 (2009).

44. T. Ishima, M. Iyo, K. Hashimoto, Neurite outgrowth mediated by the heat shock protein Hsp90α: a novel target for the antipsychotic drug aripiprazole. *Transl. Psychiatry* **2**, e170–e170 (2012).

45. H. Takeuchi, *et al.*, Hsp70 and Hsp40 improve neurite outgrowth and suppress intracytoplasmic aggregate formation in cultured neuronal cells expressing mutant SOD1. *Brain Res.* **949**, 11–22 (2002).

46. B. C. Bornhauser, P.-A. Olsson, D. Lindholm, MSAP is a novel MIR-interacting protein that enhances neurite outgrowth and increases myosin regulatory light chain. *J. Biol. Chem.* **278**, 35412–35420 (2003).

47. H. Neumann, *et al.*, Tumor Necrosis Factor Inhibits Neurite Outgrowth and Branching of Hippocampal Neurons by a Rho-Dependent Mechanism. *J. Neurosci.* **22**, 854–862 (2002).

48. M.-S. Huang, *et al.*, Roles of carbonic anhydrase 8 in neuronal cells and zebrafish. *Biochim. Biophys. Acta BBA - Gen. Subj.* **1840**, 2829–2842 (2014).

49. C. Liu, W. Ma, W. Su, J. Zhang, Prdm14 acts upstream of islet2 transcription to regulate axon growth of primary motoneurons in zebrafish. *Dev. Camb. Engl.* **139**, 4591–4600 (2012).

50. M.-S. Yoon, *et al.*, Role of phospholipase D1 in neurite outgrowth of neural stem cells. *Biochem. Biophys. Res. Commun.* **329**, 804–811 (2005).

51. Y. Zhang, Y. Kanaho, M. A. Frohman, S. E. Tsirka, Phospholipase D1-promoted release of tissue plasminogen activator facilitates neurite outgrowth. *J. Neurosci. Off. J. Soc. Neurosci.* **25**, 1797–1805 (2005).

52. M. G. Hamel, *et al.*, Multimodal Signaling by the ADAMTSs (a disintegrin and metalloproteinase with thrombospondin motifs) Promote Neurite Extension. *Exp. Neurol.* **210**, 428–440 (2008).

53. A.-M. Lepagnol-Bestel, *et al.*, SLC25A12 expression is associated with neurite outgrowth and is upregulated in the prefrontal cortex of autistic subjects. *Mol. Psychiatry* **13**, 385–397 (2008).

54. M. T. van Diepen, *et al.*, The molluscan RING-finger protein L-TRIM is essential for neuronal outgrowth. *Mol. Cell. Neurosci.* **29**, 74–81 (2005).

55. U. Wilhelmsson, *et al.*, Absence of Glial Fibrillary Acidic Protein and Vimentin Prevents Hypertrophy of Astrocytic Processes and Improves Post-Traumatic Regeneration. *J. Neurosci.* **24**, 5016–5021 (2004).

56. Y. Endo, *et al.*, Wnt-3a and Dickkopf-1 Stimulate Neurite Outgrowth in Ewing Tumor Cells via a Frizzled3- and c-Jun N-Terminal Kinase-Dependent Mechanism. *Mol. Cell. Biol.* **28**, 2368–2379 (2008).

57. G. Edwards, *et al.*, SNAIL Transctiption factor in prostate cancer cells promotes neurite outgrowth. *Biochimie* **180**, 1–9 (2021).

58. B. P. Niederöst, D. R. Zimmermann, M. E. Schwab, C. E. Bandtlow, Bovine CNS Myelin Contains Neurite Growth-Inhibitory Activity Associated with Chondroitin Sulfate Proteoglycans. *J. Neurosci.* **19**, 8979–8989 (1999).

59. H. Yamada, *et al.*, The brain chondroitin sulfate proteoglycan brevican associates with astrocytes ensheathing cerebellar glomeruli and inhibits neurite outgrowth from granule neurons. *J. Neurosci. Off. J. Soc. Neurosci.* **17**, 7784–7795 (1997).

60. S. Harashima, Y. Wang, T. Horiuchi, Y. Seino, N. Inagaki, Purkinje cell protein 4 positively regulates neurite outgrowth and neurotransmitter release. *J. Neurosci. Res.* **89**, 1519–1530 (2011).

61. C. Liu, *et al.*, Regulator of G protein signaling 5 (RGS5) inhibits sonic hedgehog function in mouse cortical neurons. *Mol. Cell. Neurosci.* **83**, 65–73 (2017).

62. S. Yin, *et al.*, RGS5 augments astrocyte activation and facilitates neuroinflammation via TNF signaling. *J. Neuroinflammation* **20**, 203 (2023).

63. D. M. Martin, *et al.*, *PITX2* is required for normal development of neurons in the mouse subthalamic nucleus and midbrain. *Dev. Biol.* **267**, 93–108 (2004).

64. O. B. Ilyinsky, *et al.*, Effects of opioid peptides and naloxone on nervous tissue in culture. *Neuroscience* **22**, 719–735 (1987).

65. I. Ahmed, Y. Calle, S. Iwashita, A. Nur-E-Kamal, Role of Cdc42 in neurite outgrowth of PC12 cells and cerebellar granule neurons. *Mol. Cell. Biochem.* **281**, 17–25 (2006).

66. M. D. Brown, B. J. Cornejo, T. B. Kuhn, J. R. Bamburg, Cdc42 stimulates neurite outgrowth and formation of growth cone filopodia and lamellipodia. *J. Neurobiol.* **43**, 352–364 (2000).

67. L. Mohiuddin, K. Fernandez, D. R. Tomlinson, P. Fernyhough, Nerve growth factor and neurotrophin-3 enhance neurite outgrowth and up-regulate the levels of messenger RNA for growth-associated protein GAP-43 and T alpha 1 alpha-tubulin in cultured adult rat sensory neurones. *Neurosci. Lett.* **185**, 20–23 (1995).

68. G. Morfini, M. C. DiTella, F. Feiguin, N. Carri, A. Cáceres, Neurotrophin-3 enhances neurite outgrowth in cultured hippocampal pyramidal neurons. *J. Neurosci. Res.* **39**, 219–232 (1994).

69. D. M. White, Contribution of neurotrophin-3 to the neuropeptide Y-induced increase in neurite outgrowth of rat dorsal root ganglion cells. *Neuroscience* **86**, 257–263 (1998).

70. C. B. Alves, *et al.*, Caffeine and adenosine A2A receptors rescue neuronal development *in vitro* of frontal cortical neurons in a rat model of attention deficit and hyperactivity disorder. *Neuropharmacology* **166**, 107782 (2020).

71. H. Maruyama, K. Fujiwara, M. Kumeta, D. Koyama, Ultrasonic control of neurite outgrowth direction. *Sci. Rep.* **11**, 20099 (2021).

72. M. Hashimoto, E. Rockenstein, M. Mante, M. Mallory, E. Masliah, β-Synuclein Inhibits α-Synuclein Aggregation: A Possible Role as an Anti-Parkinsonian Factor. *Neuron* **32**, 213–223 (2001).

73. H. J. Lee, K. Lee, H. Im, α-Synuclein modulates neurite outgrowth by interacting with SPTBN1. *Biochem. Biophys. Res. Commun.* **424**, 497–502 (2012).

74. S. Mitsuzawa, *et al.*, Reduced PHOX2B stability causes axonal growth impairment in motor neurons with TARDBP mutations. *Stem Cell Rep.* **16**, 1527–1541 (2021).

75. K. M. Munro, A. Nash, M. Pigoni, S. F. Lichtenthaler, J. M. Gunnersen, Functions of the Alzheimer's Disease Protease BACE1 at the Synapse in the Central Nervous System. *J. Mol. Neurosci.* **60**, 305–315 (2016).

76. J. Zhang, *et al.*, Sez-6 may play an important role in neurite outgrowth through the PKCgamma signaling pathways. *Z. Naturforschung C J. Biosci.* **66**, 614–620 (2011).
